# SourceSet: A graphical model approach to identify primary genes in perturbed biological pathways

**DOI:** 10.1371/journal.pcbi.1007357

**Published:** 2019-10-25

**Authors:** Elisa Salviato, Vera Djordjilović, Monica Chiogna, Chiara Romualdi

**Affiliations:** 1 IFOM - The FIRC Institute of Molecular Oncology, Milan, Italy; 2 Department of Biostatistics, University of Oslo, Oslo, Norway; 3 Department of Statistical Sciences, University of Bologna, Bologna, Italy; 4 Department of Biology, University of Padova, Padova, Italy; University of California San Diego, UNITED STATES

## Abstract

Topological gene-set analysis has emerged as a powerful means for omic data interpretation. Although numerous methods for identifying dysregulated genes have been proposed, few of them aim to distinguish genes that are the real source of perturbation from those that merely respond to the signal dysregulation. Here, we propose a new method, called SourceSet, able to distinguish between the primary and the secondary dysregulation within a Gaussian graphical model context. The proposed method compares gene expression profiles in the control and in the perturbed condition and detects the differences in both the mean and the covariance parameters with a series of likelihood ratio tests. The resulting evidence is used to infer the primary and the secondary set, i.e. the genes responsible for the primary dysregulation, and the genes affected by the perturbation through network propagation. The proposed method demonstrates high specificity and sensitivity in different simulated scenarios and on several real biological case studies. In order to fit into the more traditional pathway analysis framework, SourceSet R package also extends the analysis from a single to multiple pathways and provides several graphical outputs, including Cytoscape visualization to browse the results.

## Author summary

The rapid increase in omic studies has created a need to understand the biological implications of their results. Gene-set analysis has emerged as a powerful means for gaining such understanding, evolving in the last decade from the classical enrichment analysis to the more powerful topological approaches. Although numerous methods for identifying dysregulated genes have been proposed, few of them aim to distinguish genes that are the real source of perturbation from those that merely respond to the signal dysregulation. This distinction is crucial for network medicine, where the prioritization of the effect of biological perturbations may help in the molecular understanding of drug treatments and diseases. Here we propose a new method, called SourceSet, able to distinguish between primary and secondary dysregulation within a graphical model context, demonstrating a high specificity and sensitivity in different simulated scenarios and on real biological case studies.

This is a *PLOS Computational Biology* Methods paper.

## Introduction

The high-throughput “omics” approaches, such as genomics, proteomics, and transcriptomics, have been producing large quantities of data growing in size over time. While this information provides a more detailed knowledge of the molecular status of biological systems, at the same time it poses new challenges to the scientific community. In fact, the specification of models that can represent complex and dynamic biological systems has become a major bottleneck nowadays.

One of the most promising and widely used computational approaches for identifying dysregulated genes—typically between two conditions—is gene set analysis [1], that moves from a gene-centered perspective towards gene set-centered analysis, the gene sets being defined as functionally related groups of genes, such us, for example, *pathways* in KEGG or REACTOME [2, 3].

Among gene set-centered analyses, Topological Pathway Analysis (TPA) exploits the explicit information on biological interactions among genes pictured in a pathway to enhance and improve inferential analysis [4]. Indeed, a biological pathway can be converted into a graphical structure where nodes are genes, and edges are biochemical interactions among them [5, 6].

Most of such TPA methods compute a score for the entire pathway [7–11], others suggest a subsequent refinement of the analysis aimed at identifying subnetworks (also called modules) [12–19], which represent signal paths, consistent with the condition under study.

Most methods proposed for this task identify all components affected by the condition and do not aim to distinguish genes that are the real source of perturbation (for example, due to mutation, copy number variations, or epigenetic changes) from those that merely respond to the signal dysregulation. Adopting the terminology proposed in [20], these methods are not designed to distinguish between the *primary dysregulation* and the effect of the so-called network propagation, which is the *secondary dysregulation*, especially when the primary dysregulation is played by a gene which itself does not show a strong and statistically significant differential expression [21]. As typical examples of situations in which a dysregulation allows a distinction into primary and secondary, consider intervention studies, such as knock-in/out or drug-response studies, where the expression of one or more molecular targets is experimentally modified. But the distinction also occurs in classical case-control studies: here, the primary dysregulation is the set of factors allowing to classify units as cases or controls, which may be thought of as a set of upstream regulators. In all these cases, approaches typically identify all significantly altered pathways (or sub-pathways), but they are unable to distinguish between primary and secondary perturbations.

Identification, quantification, and the prediction of the *primary dysregulation* along with its propagation across the cellular network is of crucial importance for network medicine, where it helps to prioritize the effect of biological perturbations for further assays or therapies [22]. For this reason, a number of new methods proposed in the last decade aim to estimate and quantify primary dysregulation [22–28]. With the exception of [25], that focuses on changes in the mean and assumes no change in the covariance matrix, the remaining methods [27–30] focus on the structural changes of the underlying graphical structure.

We propose a new method for the identification of *primary genes*, based on testing simultaneously the equality of both, the mean level, and the graphical structure. The combined test broadens the range of possible perturbations and allows us more modelling flexibility; this is especially useful in real experimental situations in which little is known about the nature of the underlying perturbation.

The method is implemented in the R SourceSet package, which also contains additional statistics and graphical devices aiding the user in interpreting the obtained results.

## Materials and methods

Identifying the set of primary genes representing the primary dysregulation—the *source set* in what follows—is the purpose of the proposed method which finds its theoretical foundation in [31]. In the following, we present the key elements of our approach. A guided illustration of the estimation procedure can be found in S1 Text.

### The source set

Let *V* represent the set of genes under study, and let normal random vectors $X_{V}^{\left(1\right)}$ and $X_{V}^{\left(2\right)}$ denote their expression levels in two conditions.

**Definition 1** *We call the set D* ⊆ *V the* source set, *if*:

1. *the distribution of* $X_{D}^{\left(1\right)}$ *differs from that of* $X_{D}^{\left(2\right)}$;
2. *the conditional distributions* $X_{\bar{D}}^{\left(1\right)}|X_{D}^{\left(1\right)}$ *and* $X_{\bar{D}}^{\left(2\right)}|X_{D}^{\left(2\right)}$ *coincide*, *where* $\bar{D}=V\backslash D$.

*Furthermore*, *we say that D is a minimal source set*, *if no proper subset of it is itself a source set*.

In words, *D* contains all genes in *V* that marginally differ in distribution in the two conditions, but, conditionally on their realization, leave the distribution of the remaining genes unchanged. Thus, assuming no confounding factors, genes in *D* represent elements that may be considered as the starting point of the dysregulation process. It is only fair to emphasize that, although clear from a mathematical point of view, a biological interpretation of Definition 1 is not elementary. Indeed, pathway annotation is far from being exhaustive, and it could fail to annotate some genes. Thus, if the origin of the dysregulation is an event (an up/down expression regulation, a mutation or a ipo/iper methylation) involving a gene of the pathway which is not annotated, *D* will contain the elements of the network closest to the real (unknown) source of dysregulation.

A naive strategy to identify the set *D* from data would require testing all possible subsets of *V*, but the number of potential source sets grows with the power of *p*, making the search space too large for many practical applications. However, if graphical models are employed to represent pathways, we can take advantage of the graphical syntax to identify a set of genes which contains, if not coincides with, *D*.

### The graphical framework

We assume to model the data of the same pathway in two different experimental conditions as realizations of two Gaussian graphical models sharing the same decomposable graph *G*. Here, *G* = (*V*, *E*) is obtained from the pathway topology conversion, where *V* and *E* represent genes and biochemical reactions, respectively.

A major advantage of decomposable graphs is that they allow for a clique-grained description. Let *C*_*i*_, *i* = 1, …, *k*, be the cliques, i.e. the maximal fully connected subgraphs of the graph *G*. Let *S*_*i*_ = *C*_*i*_ ∩ *C*_*i*−1_, and *R*_*i*_ = *C*_*i*_\*C*_*i*−1_, be an associated sequence of separators and residuals, *i* = 2, …, *k*. The cliques can be arranged so that the density of any normal random vector *X*_*V*_ with the graphical structure *G* factorizes as

$$f\left(x_{V}\right)=f\left(x_{C_{1}}\right)f\left(x_{R_{2}}|x_{S_{2}}\right)\cdots f\left(x_{R_{k}}|x_{S_{k}}\right),$$ (1)

where *f* denotes a generic density function [32].

Density factorization in (1) is reflected in the decomposition of a two sample testing problem. If $X_{V}^{\left(1\right)}$ and $X_{V}^{\left(2\right)}$ denote gene expression levels in two conditions, then the global hypothesis of equality of the two distributions, $H:X_{V}^{\left(1\right)}\overset{d}{=}X_{V}^{\left(2\right)}$, decomposes according to (1) as

$$H=\overset{k}{\underset{i=1}{\cap }}H_{i},\;H_{i}\;:\;X_{R_{i}}^{\left(\text{1}\right)}|X_{S_{i}}^{\left(\text{1}\right)}\;\overset{d}{=}\;X_{R_{i}}^{\left(\text{2}\right)}|X_{S_{i}}^{\left(\text{2}\right)},\;i=1,\ldots ,k,$$ (2)

where we define *R*_1_ = *C*_1_ and *S*_1_ = ⌀.

The ordering of cliques in (1) is not unique. There are at least *k* such orderings of the cliques, one for each choice of the root clique. Let *C*_*i*1_, …, *C*_*ik*_ denote the *i*th ordering, having *C*_*i*1_ = *C*_*i*_ as the root clique, and *S*_*ij*_ and *R*_*ij*_ be a corresponding sequence of separators and residuals, *i*, *j* = 1, …, *k*. For a fixed ordering *i*, let *H*_*ij*_ denote the *j*-th local hypothesis, i.e. $H_{ij}\;:\;X_{R_{ij}}^{\left(\text{1}\right)}|X_{S_{ij}}^{\left(\text{1}\right)}\;\overset{d}{=}\;X_{R_{ij}}^{\left(\text{2}\right)}|X_{S_{ij}}^{\left(\text{2}\right)}$. The main building block of our approach is the following result that states that we can estimate the source set from data by testing hypotheses *H*_*ij*_.

**Proposition 1** *The random set* ${\hat{D}}_{G}$

$${\hat{D}}_{G}=\overset{k}{\underset{i=1}{\cap }}\underset{\left\{j:\;H_{ij}\;rejected\right\}}{\cup }C_{ij},$$ (3)

*is an estimator of the source set*.

For the proof of the above proposition, and a more detailed exposition of the theory, we refer the interested reader to S2 Text.

As outlined in the previous section, the minimal source set *D* is our quantity of interest. On the other hand, set ${\hat{D}}_{G}$ estimates the smallest source set identifiable by means of cliques and separators of the underlying graph, *D*_*G*_, that we call the *graphical source set*. In some important cases, the graphical source set will coincide with the minimal seed set. In particular, *D* = *D*_*G*_ whenever *D* coincides with a separator set in *G*. When this is not the case, the graphical source set will contain additional nodes that, from the point of view of perturbation identification, can be seen as false positives (see the scenario 3 in Simulation studies). One could then try to “drill down” and identify $\hat{D}\subset {\hat{D}}_{G}$ by performing additional statistical tests. Since determining statistical properties of such a two-step approach is far from trivial, we leave this task for future work.

Let ${\hat{D}}_{G,i}=\cup_{\left\{j:\;H_{ij}\;\text{rejected}\right\}}C_{ij}$. In our setting, set ${\hat{D}}_{G}=\cup_{i=1}^{k}{\hat{D}}_{G,i}$ contains all genes affected by the perturbation. The set ${\hat{D}}_{G}\subseteq {\hat{D}}_{G}$ represents the graphical hull of genes which can be deemed to be responsible for the dysregulation. From now on, when no ambiguity can arise, we will refer to ${\hat{D}}_{G}$ simply as the source set (or primary set), and to ${\hat{D}}_{G}\backslash {\hat{D}}_{G}$ as the secondary set.

### Estimation

Estimating source set according to (3) requires testing a collection of hypotheses {*H*_*ij*_, *i*, *j* = 1, ‥, *k*}. Although *H*_*ij*_ regards equality of conditional distributions, if log-likelihood ratio test (LLR) is used no conditional distribution needs to be estimated. Namely, if *λ*_*ij*_ denotes LLR for *H*_*ij*_, then *λ*_*ij*_ = *λ*(*C*_*ij*_) − *λ*(*S*_*ij*_), where *λ*(*C*_*ij*_) and *λ*(*S*_*ij*_) are LLR criteria for testing equality of marginal distributions induced by *C*_*ij*_ and *S*_*ij*_. Thus, to obtain ${\hat{D}}_{G}$, it is enough to compute:

$$\lambda \left(A\right)=\sum_{l=1}^{2}n_{l}\;\text{log}\frac{|{\hat{\Sigma }}_{A}|}{|{\hat{\Sigma }}_{A}^{\left(l\right)}|}$$ (4)

for *A* ∈ {*C*_1_, …, *C*_*k*_, *S*_1_, …, *S*_*k*_}, where ${\hat{\Sigma }}_{A}$ denotes a block submatrix of Σ, corresponding to the nodes in *A*, $|\hat{\Sigma }|$ is the determinant of the maximum likelihood estimate of the covariance matrix of $X_{V}^{\left(1\right)}$ and $X_{V}^{\left(2\right)}$, Σ, under *H*, ${\hat{\Sigma }}^{\left(l\right)}$ (*l* = 1, 2) the maximum likelihood estimate of Σ^(*l*)^ under the general alternative, and *n*_*l*_ the sample size in condition *l*, *l* = 1, 2.

The LLR test is well defined whenever the number of samples for the smaller group, *n* = min(*n*_1_, *n*_2_), is greater than the cardinality of the largest clique p* = max(|*C*_1_|, …, |*C*_*k*_|). Indeed, the estimates of the covariance matrices in (4) must be positive definite. In practice, high-throughput experiments are usually done with very few replicates due to budgetary constraints, which makes the LLR test applicable to a limited number of cases. To illustrate this point, Table 1 provides information about the size of the maximal clique in KEGG and Reactome pathways. For example, a data set of 14 samples in one class allows to analyze only a half of KEGG and Reactome pathways (median maximal clique size *q*_50_ in Table 1 is close to 13 for all species and both databases). Moreover, even when the number of samples is sufficient (i.e., *n* ≈ p*) and the maximum likelihood estimate exists, the sample covariance matrix can no longer be considered a good estimate of the covariance matrix.

**Table 1:** Biological pathways properties. The median (*q*_50_) and the third quartile (*q*_75_) of the distribution of (a) the cardinality of the largest clique and (b) the ratio of the number of edges of the transformed graph (decomposable, undirected graph *G*) to the number of edges of the original graph (in parentheses) for pathways in KEGG and Reactome databases. Pathway annotation is taken from the graphite Bioconductor package (version 1.28.2).

Species	KEGG					Reactome				
	*q*_50_		*q*_75_		*N*	*q*_50_		*q*_75_		*N*
H.sapiens	13	(1.58)	23	(1.97)	298	9	(1.07)	24	(1.26)	1824
M.musculus	13	(1.61)	25	(2.02)	294	9	(1.06)	22	(1.25)	1481
C.elegans	7	(1.15)	11	(1.50)	103	5	(1.01)	13	(1.25)	800

Great efforts have been undertaken to gain efficiency in (large-scale) covariance estimation with small-sample data. Among the available strategies, shrinkage methods appear to be a valuable option. See, for example [33], which is shown to enjoy certain optimality properties within the “large *p*, large *n*” asymptotics. To the best of our knowledge, however, a discussion of the best shrinking strategy and of its impact on the validity of *p*-values in the context of two-sample testing is not available in the literature.

In SourceSet, we introduce a new estimation strategy, named *TEGSmin*, based on an *ad-hoc* ridge estimator [34], that adds a small quantity to the diagonals of the covariance matrices to be estimated. Extensive simulation studies (S1 Fig) have shown that this strategy has an impact on the validity of *p*-values in the context of two-sample testing procedure by far preferable to that due the use of more standard shrinking strategies, such as those in [33]. Indeed, the estimation procedure that we adopt gives rise to *p*-values for the LLR tests whose distribution is stochastically larger than the theoretical one, meaning that we obtained a valid, although conservative, testing procedure. All details and simulations can be found in S3 Text.

### The graphical structure

As already mentioned above, the graphical structure *G* is derived from pathway structure. To this end, a pathway is first converted to a directed graph, which is then transformed into a decomposable undirected graph *G* in two steps, in graph terminology known as *moralization* and *triangulation*. Both graph operations require adding edges to the original graph and *G* typically has many more edges with respect to the original pathway graph (see Table 1). Since the presence of an edge between two genes in *G* indicates a possibility of a direct connection, the fact that *G* is highly connected implies that the restrictions imposed by the graphical structure are mild. *G* can be seen as a network featuring a variety of possible paths a signal could take, of which potentially only some are indeed active. In other words, the “true” structure could be any subset of the edges featured in *G*. This means also that the “true” structures may be different in two conditions (see the scenario 4 in Simulation studies) and in that case our method will, given sufficient statistical power, detect this dysregulation and affected genes (nodes) will be members of the estimated source set ${\hat{D}}_{G}$.

### Multiple testing correction

The procedure for obtaining ${\hat{D}}_{G}$ requires testing equality of all conditional distributions of the form $X_{R_{ij}}|X_{S_{ij}}$ (*i*, *j* = 1, …, *k*). The number of distinct tests among them depends on *G* and equals $m=k+\sum_{i=1}^{k}v\left(C_{i}\right)$, where *v*(*C*_*i*_) is the number of distinct separators within *C*_*i*_ (*i* = 1, …, *k*). This calls for multiple testing error correction. To address this problem we use two versions of the method proposed in [35], which relies on permutations to obtain the joint distribution of the *p*-values. It attenuates the well known conservativeness of the Bonferroni procedure by taking into account the dependence between *p*-values.

More specifically, when the maximum likelihood estimates are used, both asymptotic and per-hypothesis permutation *p*-values can be calculated, and max*T* and min*P* approach can be adopted, respectively. If the regularized estimates are calculated, the asymptotic distribution is no longer valid and only the min*P* version and the per-hypothesis permutation *p*-values are applicable. Note that by controlling the family wise error rate (FWER), we control the inclusion of false positives in ${\hat{D}}_{G}$. More details on min *P* and max *T* algorithm can be found in S4 Text.

The number of permutations depends on the method, the *α* level chosen, and the number of hypotheses. Although it would be best to always use the collection of all possible permutations, this is computationally not feasible even for moderate datasets. For this reason, we use a collection of randomly generated permutations, as suggested in [36]. The Authors recommend using *m*/*α* permutations as an absolute minimum for min *P*, and 1/*α* permutations for max *T*. The min *P* method usually requires more permutations than max *T*, due to the discrete nature of the permutation *p*-values.

### Workflow of the algorithm

**Fig 1:**
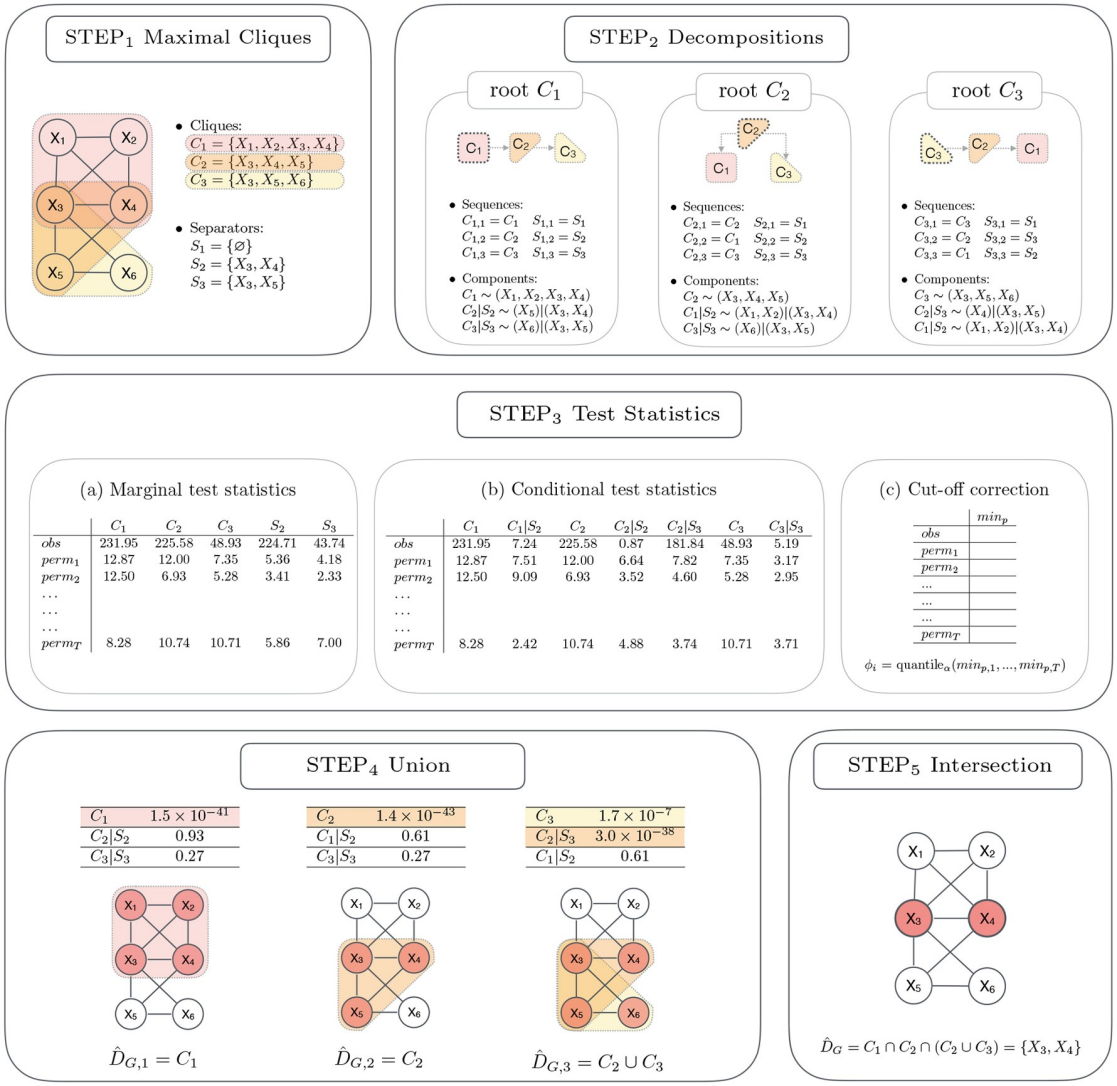
Basic workflow of the SourceSet algorithm for the analysis of a single graph.

Given a graph *G*—typically representing the dependency structure encoded in a pathway—and a matrix of sample observations—that contains the measured expression levels of the genes in the two experimental conditions—a general scheme of the procedure is described in what follows (see also Fig 1). It is worth noting that, in view of applying the multiple correction procedure, a set of permuted datasets is also prepared, to be used in some of the next steps.

1. *Maximal Cliques*: identify the set of the maximal cliques, *C*_*i*_, *i* = 1, …, *k*, and the set of separators, *S*_*i*_, *i* = 1, …, *k*, of the decomposable graph *G*;
2. *Decompositions*: list all *k* orderings *C*_*i*1_, …, *C*_*ik*_, using each clique *C*_*i*_, *i* = 1, …, *k*, in turn as the root clique;
3. *Test Statistics*:
  - *Marginal test statistics*: calculate marginal test statistics for the cliques and separators, *λ*(*C*_*i*_) and *λ*(*S*_*i*_), *i* = 1, …, *k*, for the original and the permuted datasets;
  - *Conditional test statistics*: calculate test statistics for *H*_*ij*_ as the difference between the corresponding clique and separator marginal test statistics;
  - *Cut-off correction*: control the FWER by min *P* or max *T*; find the cut-off based on the test statistics computed in the original and the permuted datasets in (b);
4. *Union*: compute for each decomposition *i*, *i* = 1, …, *k*, the quantity ${\hat{D}}_{G,i}$ by making the union of the cliques found to be significantly dysregulated;
5. *Intersection*: estimate the source set, ${\hat{D}}_{G}$, by taking the intersection over the decompositions *i*, *i* = 1, …, *k*, of the sets ${\hat{D}}_{G,i}$ obtained in the union step.

## Results

### Simulation studies

**Fig 2:**
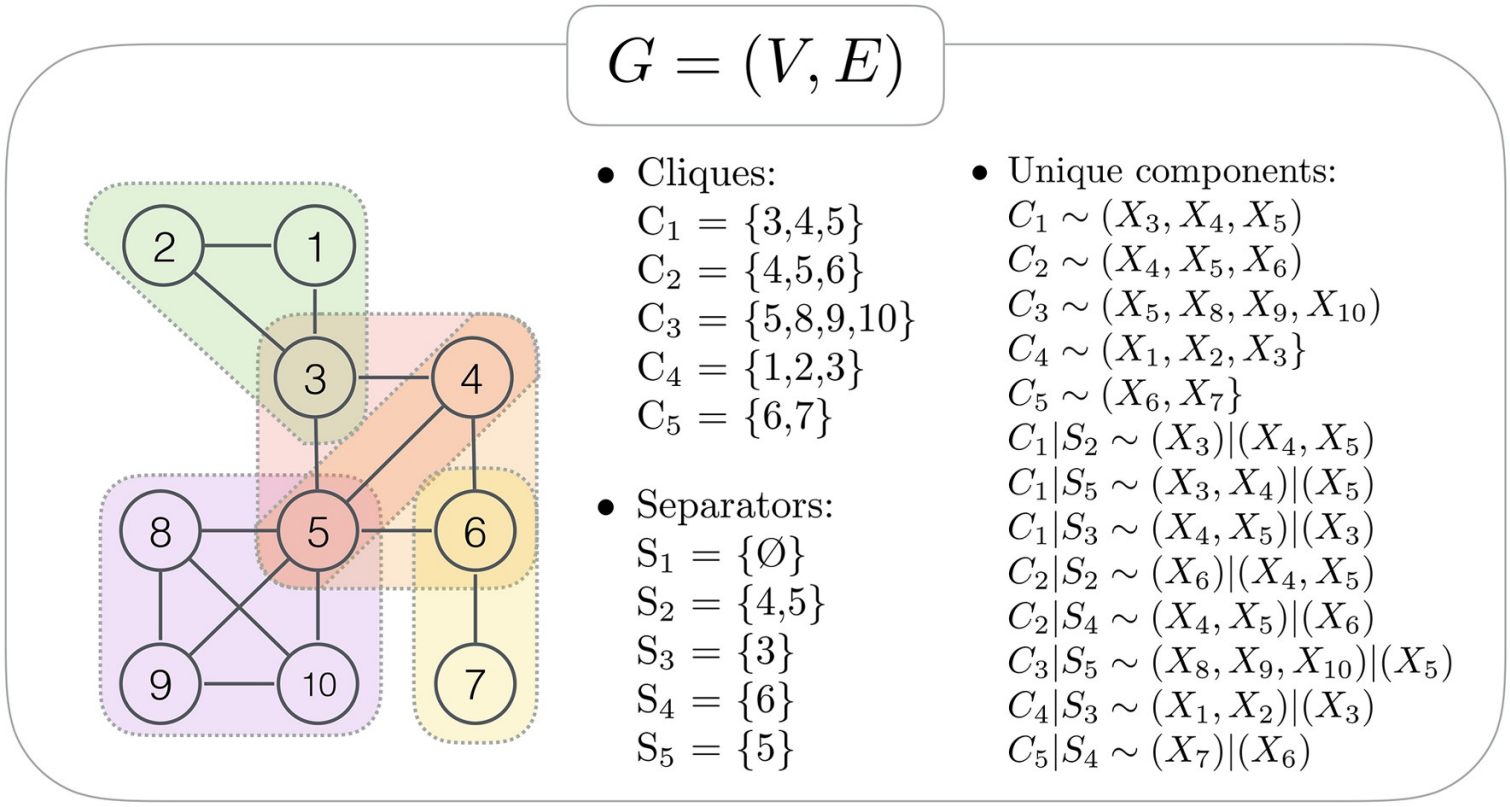
Decomposable graph used in the simulation study. Decomposable graph *G* consisting of |*V*| = 10 nodes, *k* = 5 cliques and *m* = 13 unique components.

We studied the finite sample behavior of our method through a simulation study, following the algorithm described in [37]. Data in the two conditions are assumed to be realizations of independent multivariate normal random variables *X*_*V*_, Markov with respect to the same graph *G* shown in Fig 2. Graph *G* consists of 10 nodes forming *k* = 5 cliques, with the maximum size p* = 4. Fig 2 also shows cliques of *G* and lists all *m* = 13 distinct distributions whose equality in the reference and perturbed condition is tested by our approach.

Parameters of the reference condition (the mean and covariance matrix) are set to estimates obtained from a gene set of the same cardinality as *G* randomly selected from the Acute Lymphocytic Leukemia (ALL) dataset [38]. Parameters of the perturbed condition are obtained by acting on the means and variances of the intervened variables *X*_*D*_, *D* ⊆ *V*, so as to leave the parameters of the conditional distributions $X_{\bar{D}}|X_{D}$, $\bar{D}=V\backslash D,$ unchanged. The simulation model thus assumes that the dysregulation mechanism directly affects primary gene(s), and then propagates to the remaining variables through the connections pictured in the graph.

With reference to the graph in Fig 2, we considered four different scenarios:

1. *source set is empty*, *i*.*e*., *there is no dysregulation*;
2. *the perturbation affects the mean and the variance of node 5*. *Here*, *the graphical source set D*_*G*_ *coincides with the minimal source set D* = {5};
3. *the perturbation affects the mean and the variance of node 10*. *Here*, *the graphical source set D*_*G*_ = {5, 8, 9, 10} *is larger than the minimal source set D* = {10};
4. *the perturbation affects the graphical structure and removes two edges*: *between nodes 1 and 3*, *and 2 and 3*. *The graphical source set D*_*G*_ *and the minimal source set D* = {1, 2, 3} *coincide*.

In scenarios 2 and 3, we dysregulated, respectively, node 5 and node 10 at three different levels of intensity (*mild*, *moderate*, and *strong*). These consist of an increase in the mean and the variance parameters of 20%, 60%, and 100%, respectively. In the fourth scenario, to remove the two edges, we set the corresponding elements of the inverse of the covariance matrix of the reference condition to zero.

After setting the mean and the variance parameters for the two conditions as described above, we simulated 500 datasets for each combination of a source set, dysregulation intensity, and sample size. We considered three different sample sizes (*n*_1_ = *n*_2_ = 5, 10, 25), which allowed us to calculate both the maximum likelihood and regularized estimate of the covariance matrix. All the parameters used in the simulation can be found in the SourceSet package, through the data(simulation) command.

**Fig 3:**
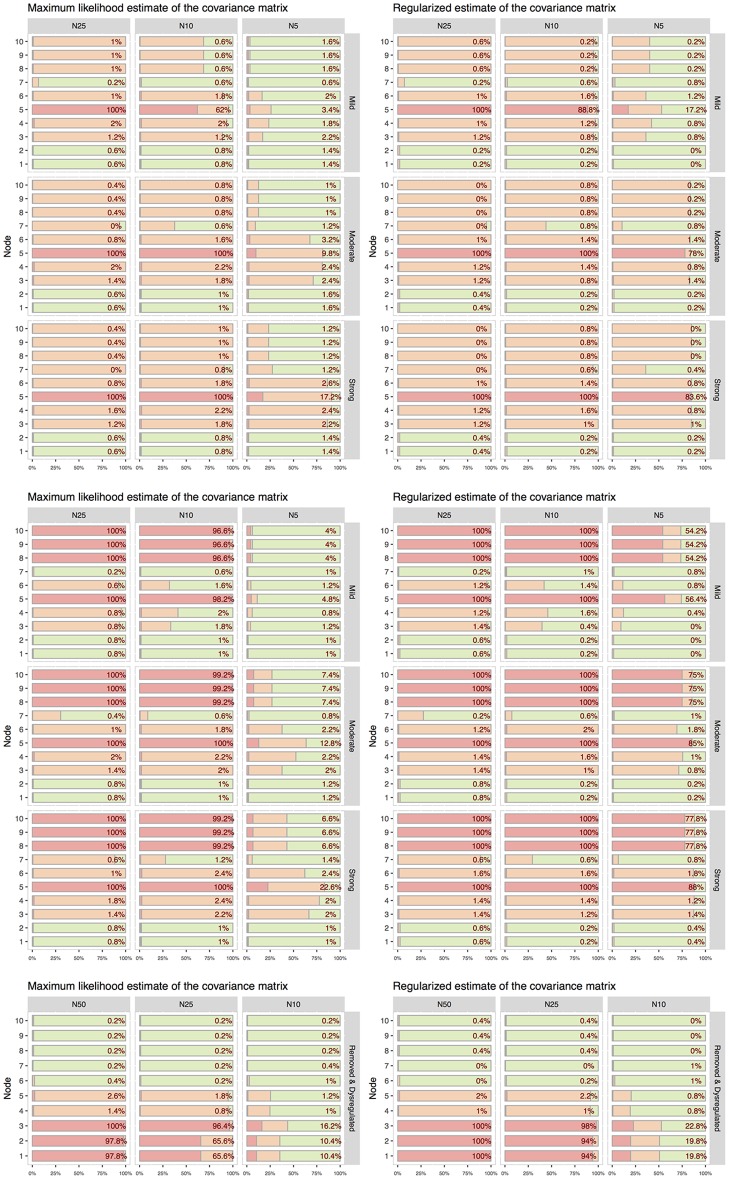
Simulation study results under the alternative hypothesis in *scenario 2* (top panel), in *scenario 3* (middle panel), and in *scenario 4* (bottom panel). On the left, results based on the maximum likelihood estimate of the covariance matrix; on the right results based on the regularized estimate. Each subpanel corresponds to a different combination of sample size (columns) and intensity of dysregulation (rows). Inside subpanels, for each node *v* ∈ *V*, a stacked bar chart shows the percentage of Monte Carlo runs in which $v\in {\hat{D}}_{G}$ (red, primary set), $v\in {\hat{D}}_{G}\backslash {\hat{D}}_{G}$ (orange, secondary set) and $v\in V\backslash {\hat{D}}_{G}$ (green). The Monte Carlo error is bounded above by 2.2%.

To evaluate the performance of our procedure, we estimated the false positive rate, i.e. the probability that a source set estimate contains a false positive (scenario 1) and the proportion of true positives (scenario 2, 3 and 4). We adopted the min *P* approach for multiple error correction and we controlled FWER at level *α* = 0.05. Results are shown in Table 2 and Fig 3.

**Table 2:** Simulation scenario 1. Fractions of misidentifications of *D* for different sample sizes and different covariance matrix estimators.

*D* = {∅}	Maximum likelihood estimate	Regularized estimate
*n*_1_ = *n*_2_ = 25	0.004	0.008
*n*_1_ = *n*_2_ = 10	0.008	0.022
*n*_1_ = *n*_2_ = 5	0.022	0.012

#### Scenario 1

Table 2 shows the Type I error of our method, i.e. the proportion of Monte Carlo runs in which the source set estimate was non-empty under the null hypothesis of no dysregulation. In this scenario, the algorithm demonstrates a very high accuracy (Type I error < 0.05) for all considered sample sizes and regardless of the choice of the method for covariance matrix estimation. The slight conservativeness is intrinsic to our estimation procedure and is especially evident under the global null hypothesis. Indeed, under the global null hypothesis at least two false rejections are needed in order to obtain a non-empty source set estimate.

#### Scenario 2 and 3

The results in the presence of a dysregulation of varying intensity in scenarios 2 and 3 are shown in the top two panels of Fig 3. At the variable level, the plot shows,for a given dysregulation strength and sample size, the percentage of Monte Carlo runs in which the variable is deemed to be a source of dysregulation, i.e. an element of the source set (red), affected by secondary perturbation, i.e. affected by the perturbation but not an element of the source set (orange), or unaffected by the perturbation (green). Results based on the maximum likelihood estimator are shown on the left; results based on the regularized covariance estimator are shown on the right.

Consider *scenario 2* shown in the top panel of Fig 3. When the dysregulation is moderate to strong, and the sample size is *n* ≥ 10, the source set is always identified correctly, i.e. ${\hat{D}}_{G}=D$. On the other hand, when the sample size is close to the theoretical limit for the existence of the LLR criterion (i.e., *n* = 5), the regularized estimator performs better and correctly identifies the source set about 84% of the times compared to the 17% reached by the maximum likelihood estimator. When the dysregulation is mild, the performances are lower for both covariance estimators, although the improvement obtained through regularization remains evident.

All the above considerations can be extended to *scenario 3* (middle panel of Fig 3), in which the graphical source set is larger than a minimal source set. As a consequence, while for the second scenario ${\hat{D}}_{G}$ coincides with *D*, in the third we are identifying the entire clique {5, 8, 9, 10}. In this case, the event $D\subset {\hat{D}}_{G}$ is of interest, coherently with the inclusion properties shown by the true graphical and non graphical source sets. It is worth stressing that the inclusion of *D* into a larger estimated gene set should not be considered a false positive in our simulation, but rather a limitation of the graphical approach (see the discussion in The graphical framework section).

#### Scenario 4

Dysregulation considered in *scenario 4* is different from the previous ones in that only the covariance parameter is affected by the perturbation. As already specified, to remove the edges between nodes 1 and 3 and 2 and 3, we set the associated elements of the inverse of the covariance matrix, i.e. concentration matrix, to zero. However, in our example these elements of the concentration matrix were already low in the reference condition, making the perturbation very mild. For this reason, in this simulation scenario we modified the concentration matrix of the reference condition by increasing the strength of conditional dependence relations (in absolute value) between nodes 1 and 3 and 2 and 3, i.e. we increased the absolute value of the associated elements in the concentration matrix. Furthermore, we considered larger sample sizes *n* = 10, 25, 50.

The results are shown in the bottom panel of Fig 3. We see that already with 25 observations, the method based on the regularized estimator achieves high detection power and correctly identifies the source set *D* = {1, 2, 3} approximately 95% of the times. Maximum likelihood estimator achieves similar performance with 50 observations. Interestingly, when *n* = 25, maximum likelihood estimator manages to identify node 3 as an element of the source set, but in two thirds of the cases (approximately 65% of times) is unable to detect nodes 1 and 2.

#### Sensitivity analysis

In many practical applications, data can be far from normally distributed. For example, they can be discrete, possibly showing a large number of zeros, or come from skewed distributions. A popular choice for adapting the non Gaussian data to the the Gaussian assumption relies on data transformation. This approach can work well in some circumstances. For example, microarray data are typically Gaussian on a log scale. Unfortunately, it can be also ill-suited in some circumstances. For example, the distributions of log counts or RPKM/FPKM in next-generation sequencing, are known to be characterized by extreme outliers, possibly leading to wrong inferences.

To explore sensitivity of our method to the presence of outliers and asymmetry, we performed analyses in *scenario 2* and *scenario 3* using data generated from two multivariate skew-normal distributions. To this aim, we used the previous means and covariance matrices for the two conditions, and considered an additional vector of skewness parameters. To mimic real applications, the skewness parameter was set to a value estimated from a real dataset. Indeed, skew normal distributions can be considered a good fit for RNA-seq log transformed expression profiles, as highlighted in the figure of S6 Text. In particular, we injected skewness in the distribution of 4 out of 10 variables (more details in S6 Text). This choice aims to reproduce realistic behavior while preserving the conditional independence structure encoded in *G* (Fig 2). Our findings, reported in S1 Fig, indicate that the SourceSet appears to be robust to the presence of outliers and skewness, giving results comparable with those obtained using multivariate normal distributions in both considered scenarios.

#### Additional simulation studies

It is interesting to investigate whether previous conclusions can be extended to larger and more complicated graphs. To this aim, we considered the *Proteoglycans in cancer* pathway (see Run-time analysis), in which we perturbed the mean level of the *fibronectin 1* gene (FN1) that appears to be the most connected node (80 neighbours). We adopted the simulation strategy described in *Comparison with other methods* section, and we considered moderate and strong dysregulation intensities for several sample sizes (*n* = 15, 35, 50). As FN1 is the unique element of at least one separator, the graphical source set coincides with the minimal source set (scenario 2). For each setting we used both the regularized and—when it existed—the maximum likelihood estimate of the covariance matrix.

S2 Fig shows the results averaged over 50 Monte Carlo runs for the 13 genes (out of the original 202) that appear in at least one source set estimate in any of the considered settings. As expected, when the regularized estimate is used, the perturbed node is the only element of the source set with a probability close to one, except for the milder configuration (*N* = 15 and moderate perturbation). Conversely, this is true for the maximum likelihood estimate only in the strongest signal setting (*N* = 50 and strong perturbation).

### Implementation

The presented method is implemented in an R package called SourceSet (CRAN repository). In particular, the method has been extended to fit into the more traditional TPA framework, where the interest is in considering more than one pathway at a time.

The SourceSet package consists of six core functions (Table 3) that, given a list of pathways to be analyzed (input pathways) and a gene expression matrix, allow the user to:

1. identify a source set and a secondary set of each graph;
2. pool results from single-pathway analyses to gain a global view of results and obtain replicable summaries of research findings through additional visualization tools and statistics;
3. connect with Cytoscape software environment [39] to visualize, explore and manipulate chosen pathways in a dynamic manner.

**Table 3:** SourceSet package main functions.

Function	Description
sourceSet	Main function
infoSource	Return a summary of the results focusing on either variables or graphs
easyLookSource	Summarize the results through a heatmap
sourceSankeyDiagram	Summarize the results through an interactive Sankey diagram
sourceCytoscape	Vizualize in Cytoscape a collection of the analyzed graphs highlighting the interesting findings
sourceUnionCytoscape	Visualize in Cytoscape the graphical union induced by the source sets of the collection of the analyzed graphs

Although the interpretation of the source set procedure for a single graph is intuitive, the global analysis of results coming from a collection of overlapping pathways can be challenging. To tackle this task, we introduce some new indices aimed at pointing the user to the most interesting genes. In detail, for each gene, we introduce three new indicators, named *relevance*, *primary.impact* and *score*, defined as follows:

- *relevance*: percentage of input graphs, such that the given gene belongs to their estimated source set, with respect to the total number of input graphs;
- *primary.impact*: percentage of input graphs, such that the given gene belongs to their estimated source set, with respect to the total number of input graphs in which the gene appears;
- *score*: a number ranging from 0 (no significance) to + ∞ (maximal significance) computed as the combination of the *p*-values of all components (of all the input pathways) containing the gene.

Ideally, genes responsible for the primary dysregulation will be elements of the source set in all input pathways that contain them and will thus have high values of *primary.impact* and *score*. However, if a given gene appears in a single pathway, and belongs to its source set, these indices can be deceptive. For this reason, *relevance* serves to identify genes that apart from being good candidates for primary perturbation, also appear frequently in the input graphs. Which index is to be preferred depends on the objective of the analysis: in case of exploratory analysis, we suggest to rely on *relevance* (see, for example, Real Data section, Case study 2).

It should be stressed that the notion of the source set is relative to a single graphical structure *G* = (*V*, *E*). The union of source set estimates of different graphical structures, i.e. pathways, is not necessarily the source set for the pooled set of genes. For example, if the only gene causing the primary dysregulation is not annotated in a given pathway, then the associated source set estimate is likely to contain genes affected by the causal gene. The only way to ensure that the global source set estimate contains only primary genes is to consider a global graph representing the graphical union of the entire collection of pathways. This possibility is given to the users by allowing them to provide the preferred input graph in the SourceSet package. However, in many cases—such as when considering unions of the KEGG and the REACTOME pathways—this strategy leads to an almost fully connected graph, which nullifies the usefulness of the biological annotations and of the proposed approach.

Some other notes on the extension to the case of multiple pathways and on the issues here discussed can be found in S5 Text. A basic introduction on the usage of the package and its features is given in S7 Text and in the vignette of the package.

#### Run-time analysis

The run-time analysis of sourceSet is nontrivial. The performance of the algorithm mainly depends on the size of the input data (the two sample sizes and the number of nodes/genes), as well as on the graphical structure. To evaluate the scalability and efficiency of the algorithm as sample size and graph complexity increase, we conducted an empirical analysis of the execution time.

**Fig 4:**
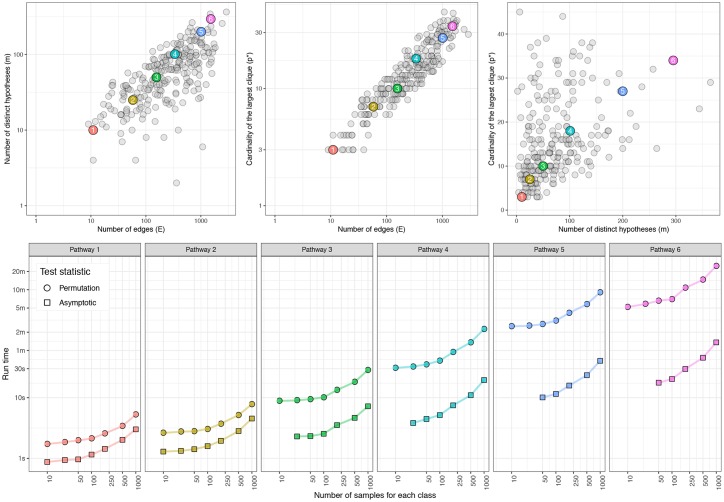
SourceSet run-time analysis. **(Top panel)** All pairwise relationships between the parameters that define the complexity of a graph (i.e., the number of edges, the number of distinct hypotheses and the cardinality of the largest clique) for 248 KEGG pathways. Six pathways—highlighted with filled circles of different colors—of increasing complexity were chosen for the run-time analysis (see also Table 4). **(Bottom panel)** Run-time for the six pathways as a function of the sample size. Permutation tests and asymptotic tests are plotted with circles and squares, respectively.

The complexity of a graph is closely related to its size and its degree of connectedness, that in our framework can be described by three parameters: the number of edges (*E*) of the graph, the number of distinct hypotheses *H*_*ij*_ to be tested (*m*), and the cardinality of the largest clique (p*). We computed these three quantities for all KEGG pathways (*N* = 248, graphite package version 1.24.1) and the results are shown in the first row of Fig 4. We then selected six of these pathways with an increasing level of complexity to be used as graphical structures in our analysis (Fig 4 and Table 4).

**Table 4:** Properties of the six pathways selected for the run-time analysis. The number of genes (|*V*|), the number of edges (*E*), the cardinality of the largest clique (p*),the number of distinct hypothesis (*m*), and the number of permutations used for the computation of the permutation *p*-values (*N*_*p*_), for the six KEGG pathways highlighted in Fig 4. Pathway annotation is taken from the graphite Bioconductor package (version 1.24.1).

Pathway name	|*V*|	*E*	p*	*m*	*N*_*p*_
1. Arrhythmogenic right	10	11	3	10	1000
2. Terpenoid backbone biosynthesis	21	58	7	25	1000
3. Platinum drug resistance	39	156	10	50	2000
4. Progesterone-mediated oocyte maturation	87	338	18	101	4040
5. Apoptosis	131	1014	27	200	8000
6. Proteoglycans in cancer	202	1516	34	295	10000

For each of the six graphs, six different sample sizes were considered *n* = 10, 50, 100, 250, 500, 1000. For each combination of the graph and sample size we generated 50 datasets under the null hypothesis of no dysregulation, and whenever the sample size allowed, in addition to the permutation test (filled circles in Fig 4), we also considered the asymptotic test for *H*_*ij*_ (filled squares in Fig 4). The execution time of the sourceSet function has been measured with microbenchmark R package.

As can be seen in Fig 4, the running time varies from a few seconds to a few dozen minutes, and grows linearly (in the original scale) with sample size and graph complexity. The increase in the graph complexity mainly affects the number of permutations necessary for the calculation of the permutation test statistic. Since the number of permutations is for computational reasons limited to 10000, complicating the graphical structure further does not significantly affect the running time. In fact, this simulation study allowed us to explore the entire range of permutations allowed by the algorithm (see Table 4).

When *H*_*ij*_ is tested with an asymptotic LLR test, permutations are used only to correct *p*-values for multiple testing. In that case, the number of permutations is lower (in our simulation equal to 500), reducing the execution time by an order of magnitude (the execution time is in the range of two seconds to two minutes). It should be emphasized that, although not implemented in this version, the algorithm is fully parallelizable. Both the estimation of the source set for multiple input graphs and the calculation of the permutation test statistics for a single graph can be run in parallel.

### Real data

In this section, different real datasets have been used to validate and illustrate the value of our method. We have considered three intervention studies and three classical case-control studies. Clearly, intervention studies offer the best possibility for the biological validation of our approach in terms of identifying the origin of perturbation and providing new biological insights. On the other hand, interpreting the results of case-control studies is more challenging since we are further away from the ground truth regarding the original perturbation: these examples should be viewed as an illustration of the wider applicability of the method.

In all following analyses, FWER is controlled at level *α* = 0.05. For any additional parameters the default settings provided by the SourceSet package were used.

#### Validation study 1: Silencing of STAT3

The High-Grade Glioma (HGG) is the most common and lethal brain tumor in humans. The over-expression of a mesenchymal gene expression signatures (MGSE) is associated with a poor prognosis, and Carro *et al*. [40] identified six transcription factors (TF) that control the expression of > 74% of the MGSE genes. Among them, STAT3 emerges as one of the master regulators and, to further investigate its role, the authors silenced STAT3 in human cells.

We downloaded pre-processed data (variance stabilized and robust spline normalized) from GEO portal (GSE19114). Gene probes were annotated using Illumina HumanHT12v3 annotation and duplicated Entrez IDs were averaged for each sample. The dataset includes 22 samples (11 knock-down and 11 control cells) and 19292 gene expression levels. The number of differentially expressed genes between the two groups (EBayes test [41], adjusted *p*-value ≤ 0.05) is 1029, and STAT3 appeared to be the most significant one (*p*-value < 0.001, log.FC = 1.301, rank = 1). Here, the exact source of perturbation is known and we expect that all pathways with STAT3 have a non-empty source set that includes STAT3.

**Fig 5:**
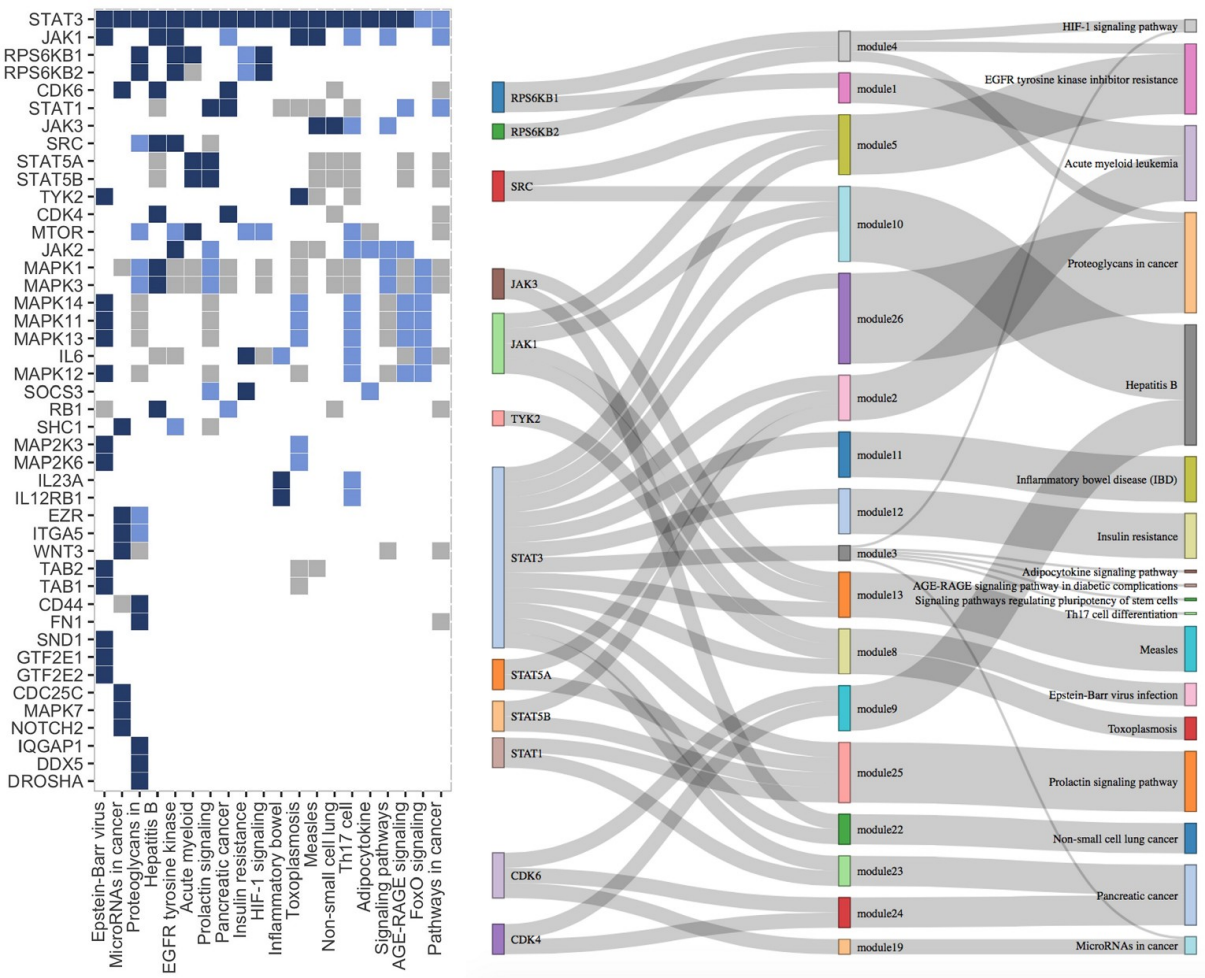
Visual summary of the source set analysis results for the STAT3 dataset. **(Left)** KEGG pathways containing STAT3 and all genes appearing in at least one estimated source set are cross-tabulated. The color of the cell (*i*, *j*) shows the relation between the *i*-th gene and the *j*-th pathway: *blue* if the gene belongs to ${\hat{D}}_{G}$ (primary source set), *light blue* if it belongs to ${\hat{D}}_{G}\backslash {\hat{D}}_{G}$ (secondary set), *grey* if the gene is participating in the considered pathway, and *white* otherwise (*i*-th gene does not belong to *j*-th pathway). **(Right)** This plot features KEGG pathways containing STAT3 and having a non-empty estimated source set, as well as all genes appearing in at least one estimated source. The three levels are to be read from left to right. A link between left element *a* and right element *b* must be interpreted as *a* ⊆ *b*. A module is defined as a subset of a source set belonging to a connected subgraph of the associated pathway.

As expected, focusing on the subset of 20 pathways containing STAT3, the silenced gene is present in the source set of all of these pathways, except for *Pathway in cancer* and *FoxO signaling* that show an empty source set (Fig 5 and S1 Table). Furthermore, in 4 out of 18 pathways, STAT3 is the only element of the source set, although secondary dysregulation involves many more genes. *Th17 differentiation* pathway provides the most obvious example: SourceSet recognizes STAT3 as the primary source of the dysregulation while classifying the remaining 39 genes as perturbed by the effect of signal propagation. Even considering the analysis of the entire KEGG collection, STAT3 emerges as the gene with the highest absolute relevance, and the fourth score (counting only genes that appear in more than one graph, see S3 Fig).

As STAT3 is not annotated in all pathways, some genes might indirectly capture the silencing effect and result as primary genes. This behavior can be observed in the graphical union of all subgraphs induced by the source set elements of KEGG pathways (S4 Fig).

#### Validation study 2: CREB knock-down

cAMP Response Element Binding Protein (CREB1) is a TF known to be overexpressed in acute myeloid and leukemia cells. Pellegrini *et al*. [42] tried to identify a panel of its potential direct targets comparing CREB1 knock-down and control cell lines.

We downloaded raw data from the GEO portal (GSE12056). Loess normalization and robust multi-array average (rma) background correction were performed according to affy package (version 1.60.0). Gene probes were annotated using Affymetrix Human Genome U133 Plus 2.0 Array data and duplicated Entrez IDs were averaged for each sample. The dataset includes 20 samples (10 human leukemia cell lines K562 with CREB1 knocked-down and 10 K562 control cells) and 22.410 gene expression levels. The differential expression analysis (EBayes test, adjusted p-value ≤ 0.05) identified 4.026 genes significantly involved in the comparison and among them we found CREB1 (p.val < 0.001, log.FC = 0.858, rank = 116). As for validation study 1 we expect CREB1 to be an element of the source set in all the pathway that contain the knock-down gene.

As reported in S2 Table, CREB1 is responsible for primary dysregulation in 92% (24 out of 26) pathways in which it is annotated and, considering the results on the entire KEGG collection it is ranked on the top of relevance (rank = 5) and score (rank = 35) lists (S3 Fig), along with several elements of the CREB family (S5 Fig).

#### Validation study 3: ABL/BCR chimera

This is the well known benchmark dataset on the ABL/BCR chimera in acute leukemia patients ALL (ALL Bioconductor package) [38]. Expression values were normalized according to rma and quantile normalization. Genes were annotated using Affymetrix Human Genome U95 Set data and duplicated Entrez IDs were averaged for each sample. Two groups of ALL patients with and without ABL/BCR genomic rearrangement (37 and 42 patients, respectively), are compared. 159 out of 8.595 analyzed genes (EBayes test [41], adjusted p-value ≤ 0.05) resulted as involved in the comparison. Among the two chimera genes only ABL1 (p.val < 0.001, log.FC = -0.634, rank = 3) reached the significance threshold (BCR: p.val = 0.114, log.FC = 0.272, rank = 250). Given the presence of the chimera we expected that i) all pathways including BCR and/or ABL1 genes will have a source set, and ii) the chimera genes will be included in the source set and that iii) the source set of *Chronic myeloid leukemia* (i.e., the pathway that describes the impact of the fusion genes in the cell) will be composed of only chimera genes.

**Fig 6:**
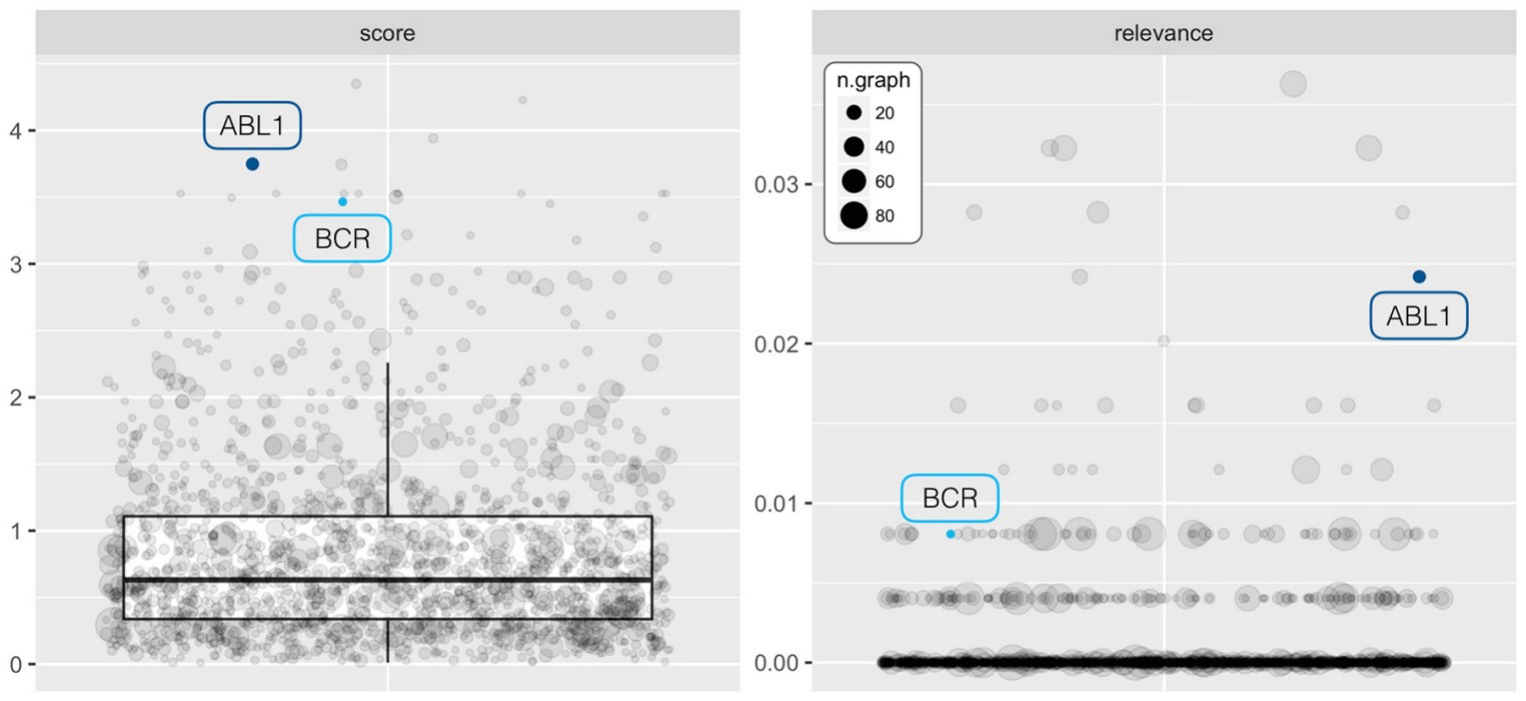
SourcSet analysis results for the chimera case study. Boxplots of score (left panel) and relevance (right panel) indices for genes annotated in at least two pathways of the whole KEGG collection (*N* = 248). The size of each point is proportional to the number of pathways in which the associated gene is annotated. ABL1 and BCR (i.e., chimera genes) are highlighted with blue and light blue dots, respectively.

As reported in the S3 Table SourceSet is able to meet all these expectations. Moreover, in comparison with all genes annotated in KEGG pathways, ABL1 and BCR appear to be among those with the best score and relevance indices (Fig 6).

Unlike the knock-out experiments, in this case the exact source of the difference is unknown, and many more genes might be directly or indirectly involved in the phenotype. A useful way to identify other genes that can interact with chimera genes and/or play a role in ALL is to observe the union of the sub-graphs induced by the primary genes of all considered pathways (S6 Fig).

#### Case study 1: Prostate cancer

Prostate cancer is one of the most frequently diagnosed malignancy and the second leading cause of cancer mortality in men. Here we used a selection of the dataset of GSE6956 [43] to compare 18 primary prostate tumors vs. 18 healthy unpaired tissues. Cancer samples were selected in order to be comparable to healthy ones in terms of ethnicity and smoke habits.Pre-processing steps of Validation study 2 were applied.

A specific KEGG pathway annotation exists for this cancer (*hsa:05215*) thus, even analyzing the entire KEGG collection, we expect to identify a significant set of primary genes characterized by the highest relevance and/or score within the target pathway.

**Fig 7:**
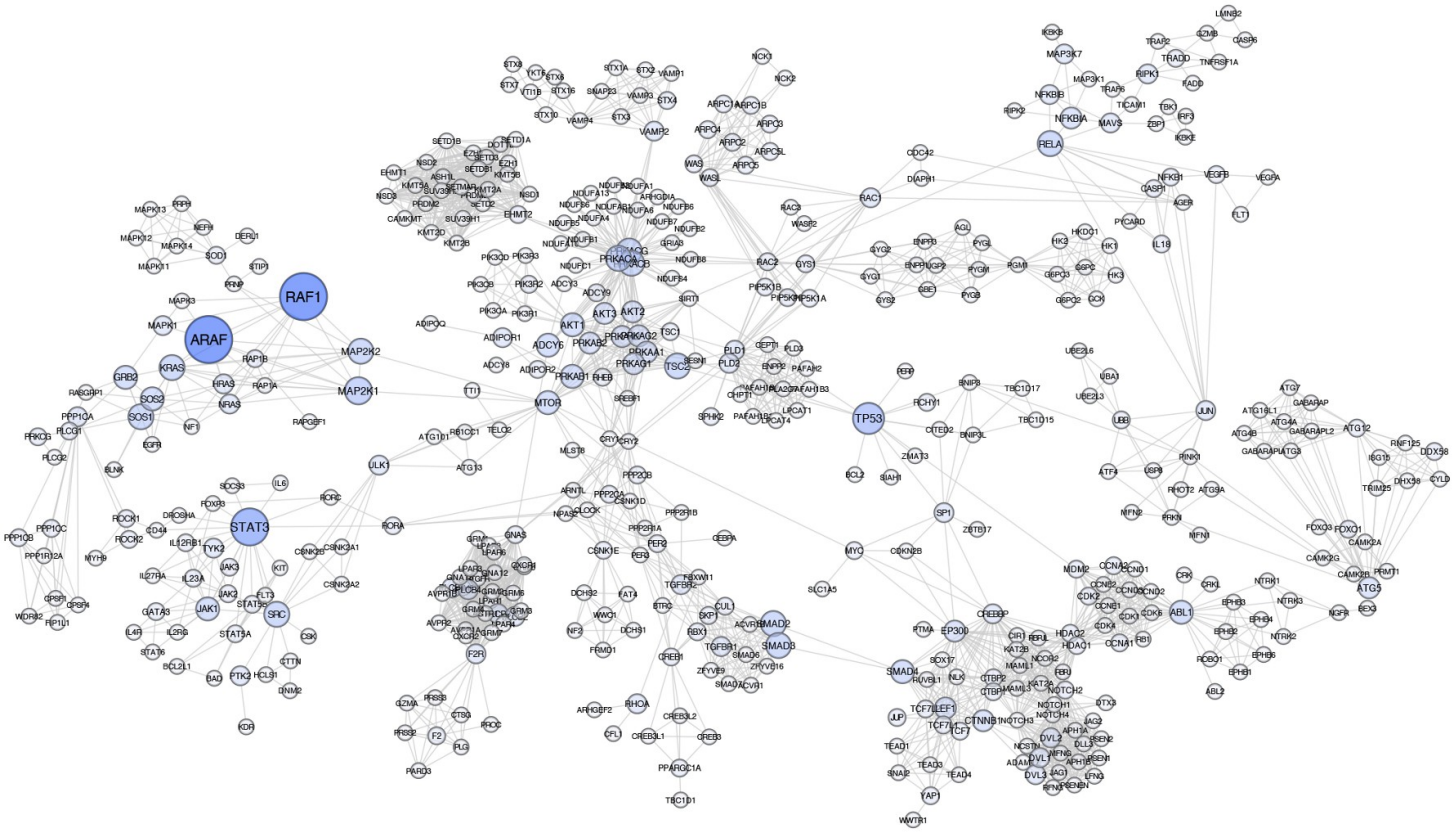
Source set analysis results for the prostate cancer study. The plot shows the main cluster of the graphical union of the source sets of the analyzed pathways, obtained through sourceUnionCytoscape function (Cytoscape version 3.6.1). The size of each node is proportional to the number of times the gene appears in a source set. The color is associated to the relevance index: higher values are indicated by dark blue colors.

The analysis was performed on 248 KEGG pathways. SourceSet identified 171 pathways (69%) with a non-empty source set (median size of six) and globally provides a panel of 869 primary genes. Interestingly, the two genes with the highest relevance are ARAF and RAF1 (Fig 7): both belong to the *Prostate Cancer* KEGG pathway. Other genes with highly attractive characteristics are STAT3, TP53 and MAP2K1 (S4 Table). The target pathway is composed of 81 genes, of which 37 marginally significant and six representing the primary dysregulation (ARAF, RAF1, HSP90AA1, HSP90AB1, AR, HSP90B1).

#### Case study 2: Pancreatic cancer

Pancreatic Ductal Adenocarcinoma (PDAC) is a very aggressive disease resistant to conventional and targeted therapeutic agents. Several studies have been performed to better understand the molecular mechanism of its evolution. Here we use a subset of GSE15471 dataset [44] to compare unpaired tissues (tumors, *n* = 20, vs healthy, *n* = 16). Pre-processing steps of Validation study 2 were applied.

Among the 20502 measured gene expression levels about 66% result as differentially expressed (Ebayes test, adjusted *p*-value ≤ 0.05). As in the previous example, a specific KEGG pathway for pancreatic cancer exists (*hsa:05212*), and we expect to find significant primary genes with high relevance and score within this pathway.

We ran SourceSet on 248 KEGG pathways. All analyzed pathways have a non-empty source set (median size of 32) for a total of 3166 primary genes. As in the case study 1, the genes with the highest relevance and score are involved in the pancreatic cancer pathway. In particular 14 out of top 20 genes ordered by relevance, belong to its source set (Table 5, left).

**Table 5:** infoSource summaries for the top 20 genes ordered by relevance index for the two pancreatic cancer studies. Number of analyzed pathways in which the gene belongs to the primary dysregulation (n.primary), number of the analyzed pathways in which it is annotated (n.graph), and its score and relevance indices; adjusted *p*-values for Ebayes test (p.value). Genes that belong to the *pancreatic cancer pathway* are marked with a star. Genes ranked in the first 20 positions in both studies are highlighted in gray.

GEO ID	GSE15471 [44]							GSE1651 [45]						
Rank		Symbol	n.primary	n.graph	score	relevance	*p*.value		Symbol	n.primary	n.graph	score	relevance	*p*-value
1	⋆	MAPK1	59	84	1.671	0.239	0.956	⋆	MAPK9	26	50	3.647	0.104	0.008
2	⋆	MAPK3	60	83	1.444	0.239	0.388		PLCB1	25	41	2.103	0.104	<0.001
3	⋆	MAP2K1	55	63	3.964	0.218	<0.001		PLCB4	25	41	2.046	0.104	0.082
4	⋆	KRAS	53	59	4.325	0.215	0.004		PLCB3	25	41	2.035	0.104	0.082
5	⋆	HRAS	49	54	4.646	0.200	<0.001		ADCY6	26	38	4.255	0.103	0.006
6		NRAS	48	54	4.643	0.196	0.001		ADCY9	25	37	4.348	0.101	0.001
7		MAP2K2	48	53	3.961	0.190	0.002		ADCY1	25	37	3.324	0.101	0.001
8	⋆	RAF1	46	57	1.304	0.186	0.112		ADCY3	24	36	4.086	0.100	0.961
9	⋆	AKT3	44	69	3.541	0.179	<0.001		PLCB2	25	43	1.935	0.099	0.656
10	⋆	AKT1	44	69	3.447	0.179	0.022	⋆	KRAS	24	59	2.447	0.098	0.002
11	⋆	AKT2	42	69	2.929	0.171	0.023		ADCY5	24	40	3.413	0.096	0.006
12		PRKACG	40	53	1.489	0.159	<0.001		ADCY8	22	36	3.052	0.092	0.312
13	⋆	MAPK9	37	50	3.394	0.148	0.001		PRKACG	23	53	1.007	0.091	0.057
14	⋆	RELA	35	51	1.725	0.144	0.014		PRKACB	23	53	0.958	0.091	0.799
15	⋆	PIK3CA	35	73	2.667	0.139	<0.001		PRKACA	23	53	0.916	0.091	0.691
16		PRKACB	35	53	1.425	0.139	0.099		ADCY7	22	34	4.238	0.091	0.021
17		PRKACA	35	53	1.194	0.139	0.081		ADCY4	22	35	4.057	0.088	0.324
18	⋆	PIK3R2	34	72	2.512	0.137	0.007		ADCY2	22	35	2.706	0.088	0.873
19	⋆	PIK3R3	33	71	2.649	0.135	0.321	⋆	MAPK10	20	49	2.580	0.082	0.386
20	⋆	PIK3R1	33	71	2.612	0.135	0.055		HRAS	19	54	2.514	0.077	0.270

Moreover, we performed the source set analysis on an independent study on pancreatic cancer [45] with a comparable number of samples (20 tumors vs. 16 healthy unpaired tissues) and the same Affymetrix platform (GPL570). Also in this dataset (GSE16515), the majority of genes are differentially expressed (43%, Ebayes test, adjusted *p*-value ≤ 0.05), and globally 2066 are primary genes (96% pathways with a non-empty source set, median size of 17). Interestingly, the results of the two studies seem to be consistent: 6 genes are shared within the first top 20 ranked genes (Table 5), and in particular, KRAS and MAPK9 are both elements of the *pancreatic cancer* pathways.

#### Case study 3: Castration resistant prostate cancer

Androgen Deprivation Therapy (ADT) suppresses the growth of prostate cancer via blockade of testicular androgen production. Despite the initial response, for a significant proportion of affected individuals, cancer cells develop castration resistance (CRPC) due to an aberrant androgen receptor (AR) expression and activation of intra tumoral androgen biosynthesis. Knuuttila et al. [46], tried to investigate the mechanism of the androgen-dependent growth of CRPC comparing tumors treated with a new therapeutic strategy (enzalutamide, *n* = 14) and control samples (vehicle, *n* = 15).

RNA-seq raw profiles were downloaded from the GEO portal (GSE95413). One low quality experiment was removed (enzalutamide tumor treated, id = MOV_25). Genes with at least ten counts in more than 75% of samples were considered for the downstream analysis, resulting in 11617 expression profiles. Transcripts per million were normalized via median ratio method (DESeq2 package) and transformed according to *log*_2_(*count* + 1). In the comparison between vehicle and enzalutamide tumors, 1204 genes (10%) resulted differenially expressed (DESeq analysis, adjusted *p*-value ≤ 0.05).

We ran SourceSet on the entire collection of KEGG pathways (*n* = 248). Among these, 84 pathways (34%) had a non-empty source set, leading to a total of 329 genes identified as primary in at least one of them. Most of the top ranked pathways are metabolism and biosynthesis related (S5 Table). In particular, *Sphingolipid metabolism* is the pathway with the highest number of primary genes (n.primary = 20) and is already reported to have a key role in several pathological processes, as well as in the resistance to treatment [47]. Investigating the five top ranked genes in S6 Table, we identified some classic androgen-regulated genes involved in CRPC, such as HSD17B6 [48], GHR [49], HLA-DMB [50], together with potentially novel biomarkers, such as GRIA2 and COMT.

### Comparison with other methods

Recently, many methods for detecting genes driving the difference between two conditions have been proposed. The method proposed in [23] searches for genes responsible for large topological changes in the gene network, i.e. regulator genes whose connections with other genes are significantly different between two conditions. [24] adapted this algorithm to single cell RNA-Seq data. The algorithm proposed in [25] is similar to the previous two, but considers covariance networks, instead of conditional dependence networks. These methods do not seem to be directly comparable with the SourceSet, since they differ in the definition of the genes driving the difference between two conditions. Namely, when the network structure is perturbed, the above methods will search for the genes most affected by the structural changes; SourceSet will flag as significant the entire portion of the network. On the other hand, the method proposed in Griffin et al. (2018) [51] is set within the Gaussian graphical framework, searches for the perturbations at the mean level, and shares some similarities with our approach. We have thus decided to compare its performance with our proposed method in a simulation study.

The method of [51] – NF in the following—is implemented in the mapggm R package, and searches for the origin of perturbation in three steps. In the first step, data from the control condition are used to estimate the graphical structure by means of a penalized regression. In the second step, the effects of network propagation are eliminated by network filtering. Finally, a set of likelihood ratio tests is performed to identify the most likely site of the original perturbation. Its *sequential* version at each step takes into account the perturbation targets identified in the previous tests. The output of the method is a list of genes, ranked according to a *p*-value for the hypothesis that the said gene is the origin of perturbation.

In this simulation study, we considered the same graph *G* on 10 nodes as before (Fig 2). We perturbed node 5, following the perturbation strategy described in [51]. Since NF searches only for the perturbations in the mean value, data in the control condition are sampled from *N*(0, Σ); in the perturbed condition from *N*(Σ*μ*, Σ), where only the fifth element of *μ*, i.e. *μ*_5_ was non-zero. We considered four different values for *μ*_5_ = 1, 5, 10, 50, corresponding to *weak*, *mild*, *moderate*, and *strong perturbation*. We also considered three different sample sizes *n* = 5, 10, 25. To render the comparison of the two methods more balanced, instead of estimating network structure encoded in Σ via penalized regression in NF, we used the prior information on the underlying graphical structure encoded in *G*. For each combination of the sample size and perturbation strength we generated 100 datasets. By allowing multiple perturbation targets, the sequential version of the NF procedure can be considered comparable to the SourceSet method in terms of power and type I error. For each Monte Carlo run, multiple testing correction (Bonferroni) was applied to the list of p-values returned by NF procedure.

Although NF was shown to be more powerful in the detection of weak perturbations, we proved that this comes at the cost of a higher type I error even in the presence of mild dysregulations (Table 6).

**Table 6:** Simulation study comparing sequential mapggm and SourceSet. Estimated power and type I error (in parentheses) for the two methods in different simulation settings. Power is computed as the probability that node 5 is identified as the origin of perturbation; Type I error is computed as the probability that at least one other node—other than node 5—is inferred to be the source of the dysregulation. A node is flagged as source of the dysregulation if its p-value is significant or it is a member of the source set estimator, for sequential mapggm and SourceSet, respectively.

	Mapggm			SourceSet		
	*n* = 5	*n* = 10	*n* = 25	*n* = 5	*n* = 10	*n* = 25
Weak	0.48 (0.81)	0.14 (0.36)	0.23 (0.17)	< 0.01 (< 0.01)	0.01 (<0.01)	< 0.01 (< 0.01)
Mild	0.84 (0.99)	1.00 (0.89)	1.00 (0.83)	0.03 (0.01)	0.38 (0.01)	1.00 (0.06)
Moderate	0.99 (1.00)	1.00 (1.00)	1.00 (0.99)	0.27 (0.01)	1.00 (0.01)	1.00 (0.02)
Strong	1.00 (1.00)	1.00 (1.00)	1.00 (1.00)	0.85 (0.02)	1.00 (0.02)	1.00 (0.03)

Indeed, even if node 5 is first-ranked in almost all the considered settings (S7 Table), NF also marks many nodes close to the true perturbation site as significant. As already noted in [51], these results imply that in more complicated configurations, such as biologically reasonable dysregulations, it might be difficult to infer which one among several top-ranked genes is the true origin of perturbation.

**Fig 8:**
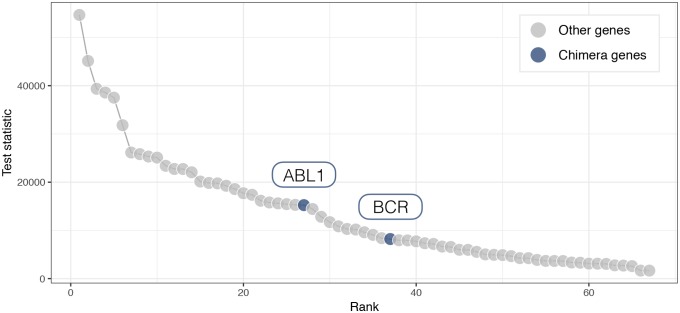
Mapggm analysis results for the chimera case study. Rank (x-axis) according to the non-sequential NF test statistic (y axis) for the 67 genes annotated in the *Chronic myeloid leukemia* KEGG pathway. ABL1 (rank = 27) and BCR (rank = 37) genes are highlighted with blue dots.

As an example, we applied NF to the dataset of the Validation study 3. We used the prior information of the *Chronic myeloid leukemia* pathway to estimate the block-precision matrix using samples of first condition (i.e., patients without ABL/BCR genomic rearrangement) and we performed gene-wise likelihood ratio tests in order to ascertain which gene was the most likely perturbation candidate. The ranking of the participating genes by the NF method is shown in Fig 8. As we can observe, most genes have very large values of test statistics and significant p-values: the two chimera genes are occupying middle ranks and would be hard to be identified as perturbation targets.

Finally, it should be stressed that NF searches for a more specific type of perturbation—the one affecting the mean only—and assumes that the covariance matrices in the control and perturbed condition are the same. As a consequence, NF is unable to detect changes at the covariance level.

**Fig 9:**
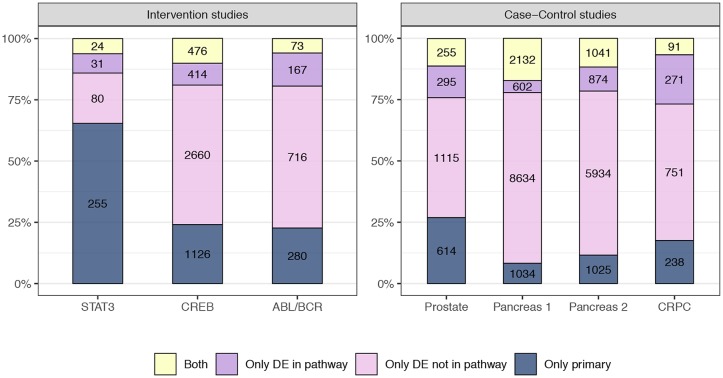
Visual summary of the results of differential expression and source set analysis. Stacked bar represents the proportion of genes flagged only by the differential expression analysis annotated in at least one KEGG pathway (violet) and not annotated in any KEGG pathway (pink), the source set analysis (blue), or both, in the comparison between the two considered conditions. Genes with *p*-values≤0.05 are flagged as deferentially expressed (DE), and those contained in the source set estimate of at least one analyzed pathway as primary.

It is also of interest to investigate how much the results of the source set analysis differ from the results of the differential analysis. Fig 9 reports the number of differentially expressed genes in the six validation and case-control studies. For each study, we report the total number of differentially expressed genes broken down into two groups according to whether they are annotated in the considered pathways. We also report the number of genes flagged by the source set analysis (those reported as primary genes), as well as the overlap between the two: the genes that are flagged both as primary, and as differentially expressed. We note that the overlap is quite limited, indicating that the two approaches bring complementary insights. Namely, some of the genes that are differentially expressed, but not primary, are downstream from the primary genes and will be flagged as secondary by the SourceSet. The genes that are flagged as primary but are not differentially expressed might be a) elements of the minimal source set whose variance and covariance patterns have been affected by the perturbation, or b) elements of the graphical source set, but not of the minimal source set.

## Discussion

We present a novel computational approach, called SourceSet, to identify primary dysregulations in perturbed pathways. Using graphical models theory, our approach detects changes in the mean expression level and in the covariance patterns and uses the resulting evidence to infer the source set, that is a set of primary genes consisting of, or closest to, the potential source of the differential behavior. We investigated the possibility that the primary genes identified by SourceSet coincide with the top ranked differentially expressed genes. Our results suggest that there is little overlap between the two lists, indicating that the two methods offer complementary insights.

The downside of the graphical approach is that it focuses on the graphical source set which might be larger than the minimal source set (see scenario 3 in Simulation studies). To tackle this issue, one may adopt a two step approach, in which the SourceSet analysis is followed by additional statistical tests on individual elements of the graphical source set, allowing to further reduce the set of possible candidates responsible for the primary perturbation. Determining statistical properties of this two step approach is not trivial, and we leave this investigation for future work.

Our method is applicable when the number of observations is far below the number of variables, a scenario that characterizes omics data. To tackle this issue, we adopted an *ad-hoc* ridge strategy for estimating the covariance matrix and a permutation approach. Simulations show that, when a dysregulation is present, SourceSet demonstrates high sensitivity and specificity in all considered scenarios, even with a low number of samples. Apart from array-based gene expression, we showed with simulated data that SourceSet can be applied to log counts or RPKM/FPKM in next-generation sequencing experiments. Other possible applications include protein abundances and metabolomic data.

A number of methods for the identification of the origin of perturbation have recently been proposed and we discuss them in Comparison with other methods section. Most of them consider different definitions of perturbations and make different underlying assumptions, which makes them applicable to more specific types of perturbations, i.e. those affecting the network structure or those affecting the mean expression. Our approach allows the perturbation to affect the mean and/or the covariance of the set of genes, broadening the range of detectable perturbations. On the other hand, when the sample size is very small or the perturbation is very weak, our empirical comparison showed that a method focusing on a more specific type of perturbation, such as [51], has more power and might be preferable.

In this work, we adopted a pathway-centered approach in which individual pathways are analyzed independently, and the results are visualized by taking the graphical union of the resulting source set estimates. To this aim, we implemented different graphical devices to guide the user in interpreting the obtained results in the SourceSet R package. Using the pathway-centered approach we analyzed three different intervention studies and we showed that SourceSet has the potential of providing new biological insights in the search for the origin of dysregulations.

It should be stressed that although the graphical union can provide valuable biological insights, it cannot be interpreted as the global source set estimate, i.e. the source set of the pooled set of genes. In order to obtain such estimate, one would need to consider a graph representing the entire set of genes under study. The drawback of the global perspective is the loss of interpretation of the biological annotation; nevertheless, this possibility is offered in the SourceSet package, where the choice of the input graph is left to the user.

## Supporting information

S1 Text A guided example. (PDF)

S2 Text Theoretical foundation of SourceSet. (PDF)

S3 Text Impact of shrinkage on *p*-values of LLR tests. (PDF)

S4 Text Multiple testing correction. (PDF)

S5 Text Some notes on the algorithm. (PDF)

S6 Text Simulation studies under the violation of the symmetry assumption. (PDF)

S7 Text SourceSet R package functions. (PDF)

S1 FigSimulation study results under the violation of symmetry for the graph in Fig 2 (main text) in *scenario 2* (top panel) and *scenario 3* (bottom panel).On the left, results based on the maximum likelihood estimate of the covariance matrix; on the right results based on the regularized estimate. Each subpanel corresponds to a different combination of the sample size (columns) and the intensity of dysregulation (rows). Inside subpanels, for each variable *X*_*v*_, *v* ∈ *V* a stacked bar chart shows the percentages of times that $v\in {\hat{D}}_{G}$ (red, primary set), $v\in {\hat{D}}_{G}\backslash {\hat{D}}_{G}$ (orange, secondary set) and $v\in V\backslash {\hat{D}}_{G}$ (green).(PDF)

S2 FigSimulation study results for the *Proteoglycans in cancer pathway* when gene *2335* is perturbated.On the top panel, results based on the maximum likelihood estimate of the covariance matrix; on the bottom panel results based on the regularized estimate. Each subpanel corresponds to a different combination of the sample size (columns) and the intensity of dysregulation (rows). Inside subpanels, for each variable *X*_*v*_, *v* ∈ *V* a stacked bar chart shows the percentages of times that $v\in {\hat{D}}_{G}$ (red, primary set), $v\in {\hat{D}}_{G}\backslash {\hat{D}}_{G}$ (orange, secondary set) and $v\in V\backslash {\hat{D}}_{G}$ (green). Only genes that appear at least one time in the source set are shown (13 out of 202). Two subpanels are missing because of the maximum likelihood estimate does not exit (i.e., *n* ≤ p*).(PDF)

S3 Fig SourcSet analysis results for STAT3 (top panel) and CREB (bottom panel) intervention studies. Boxplots of score (left panel) and relevance (right panel) indices for genes annotated in at least two pathways of the whole KEGG collection (*N* = 248). The size of each point is proportional to the number of pathways in which the associated gene is annotated. Silenced or knock-down genes are highlighted with blue dots. For more details about the interpretation of each index, see S7 Text.
(PDF)

S4 Fig sourceUnionCytoscape visualization of source set analysis results for the STAT3 study. The graphical union of all subgraphs induced by source set elements of each analyzed pathway (*N* = 248) is represented. The size of each node is proportional to the number of times the gene appears in a source set. The color is associated with the score index: higher values are highlighted with darker blue color. The number depicted on each edge represents the number of pathways in which the two genes are connected. For more details about the interpretation of each index, see S7 Text.
(PDF)

S5 Fig sourceUnionCytoscape visualization of source set analysis results for the CREB study. The graphical union of all subgraphs induced by source set elements of each analyzed pathway (*N* = 248) is represented. The size of each node is proportional to the number of times the gene appears in a source set. The color is associated with the score index: higher values are highlighted with darker blue color. The number depicted on each edge represents the number of pathways in which the two genes are connected. For more details about the interpretation of each index, see S7 Text.
(PDF)

S6 Fig sourceUnionCytoscape visualization of source set analysis results for the Chimera study. The graphical union of all subgraphs induced by source set elements of each analyzed pathway (*N* = 248) is represented. The size of each node is proportional to the number of times the gene appears in a source set. The color is associated with the score index: higher values are highlighted with darker blue color. The number depicted on each edge represents the number of pathways in which the two genes are connected. For more details about the interpretation of each index, see S7 Text.
(PDF)

S1 Table infoSource summaries for the 20 pathways in which STAT3 gene is annotated. Pathways in which the silenced gene appears in the source set are marked with a star. In particular those in which the silenced gene is the only gene of the source set are highlighted in gray. For more details about the interpretation of each index, see S7 Text.
(PDF)

S2 Table infoSource summaries for the 26 pathways in which CREB gene is annotated. Pathways in which the knock-down gene appears in the source set are marked with a star. In particular, those in which CREB is the only source set element are highlighted in gray. For more details about the interpretation of each index, see S7 Text.
(PDF)

S3 Table infoSource summary for pathways in which at least one of the chimera genes are annotated. Pathways in which ABL1 or both chimera genes appear in the source set are marked with one or two stars, respectively. In particular, those in which ABL1 and BCR are the only elements of the source set are highlighted in gray. For more details about the interpretation of each index, see S7 Text.
(PDF)

S4 Table Prostate cancer study. infoSource summary for the top five genes ordered by relevance index. Number of analyzed pathways in which the gene belongs to the primary dysregulation (n.primary) or the secondary dysregulation (n.secondary); number of analyzed pathways in which it is annotated (n.graph), and its score and relevance indices. For more details about the interpretation of each index, see S7 Text.
(PDF)

S5 Table Castration resistance prostate cancer study, top pathways. infoSource summary for the top 10 pathways, ordered by decreasing *primary.impact*. For more details about the interpretation of each index, see S7 Text.
(PDF)

S6 Table Castration resistance prostate cancer study, top genes. infoSource summary for the top five genes—annotated in at least 2 pathways—ordered by score index. Number of analyzed pathways in which the gene belongs to the primary dysregulation (n.primary) or the secondary dysregulation (n.secondary); number of analyzed pathways in which it is annotated (n.graph), and its score and relevance indices. For more details about the interpretation of each index, see S7 Text.
(PDF)

S7 Table Simulation study results for non-sequential mapggm. Precision and type I error (in parentheses) in different simulation settings. For non-sequential mapggm procedure the precision is computed as the probability that node 5 is first-ranked and significant; type I error is calculated as the probability that another node—other than node 5—is first-ranked with a significant p-value.
(PDF)
