# Supplementary material for: SourceSet: A graphical model approach to identify primary genes in perturbed biological pathways
Source: PLoS Comput Biol. 2019 Oct 25;15(10):e1007357. doi: 10.1371/journal.pcbi.1007357 (PMC6834292; doi:10.1371/journal.pcbi.1007357)
Supplement: S1 Text — (PDF) [file pcbi.1007357.s001.pdf]

S1 A guided example

Let us consider an undirected decomposable graph G, represented in (Figure S1), and a random vector $(X_1, ..., X_6)^\intercal$ with a multivariate normal distribution $\mathcal{N}_6(\mu, \Sigma)$, where $\mu \in \mathbb{R}^6$ is arbitrary, and Σ^{-1} obeys the conditional independence relations encoded in G, i.e. $\Sigma^{-1} \in \mathcal{S}^+(G)$, where $\mathcal{S}^+(G)$ is the set of symmetric positive definite matrices with zeros corresponding to the missing edges of G. Recall that two normal random variables are conditionally independent when their partial correlation coefficient – or equivalently, the associated element of Σ^{-1} – is zero. We can think of $X_1, \ldots, X_6$ as the expression level of a group of six genes, and of G as the dependency structure among these in a given pathway.

Let us now consider a perturbation of this system, caused, for instance, by epigenetic changes, by mutations or gene silencing. Let us assume that this perturbation affects X_3 and X_4, while the remaining variables – X_1, X_2, X_5, X_6 – merely respond to the perturbation of the signal. More specifically, compared to the control condition, the mean of X_3 decreases by 70%, and the pairwise correlation between X_3 and X_4 switches sign. In other words, the intervention influences the mechanism underlying the joint distribution by acting on these two variables, but leaves the conditional distribution of the remaining variables unaltered (Figure S2c). Such a perturbation will impact all marginal distributions, both the mean and the variance matrix, as illustrated in (Figure S2b) and (Figure S2a), and thus, if we employ any standard approach for detecting differential expression, we would conclude that the condition under study affects all considered genes. Although correct, this point of view fails to identify the special role of genes X_3 and X_4, which are the source of the difference between the two conditions. The notion of the source set formalizes this idea. If V is the set of genes under study, and $X_V^{(1)}$ and $X_V^{(2)}$ are the expression levels of these genes in the two conditions under study, we call the set $D \subseteq V$ the source set, if:

1. the distribution of $X_D^{(1)}$ differs from that of $X_D^{(2)}$;

2. the conditional distributions $X_{\bar{D}}^{(1)}|X_D^{(1)}$ and $X_{\bar{D}}^{(2)}|X_D^{(2)}$ coincide, where $\bar{D} = V \setminus D$.

We aim to estimate the source set from data.

Our approach starts by considering the global hypothesis of equality of the two distributions

$$H : \Sigma^{(1)} = \Sigma^{(2)} \text{ and } \mu^{(1)} = \mu^{(2)},$$

Figure S1: Decomposable graph G consisting of six nodes ($|V|$) and three cliques (k).

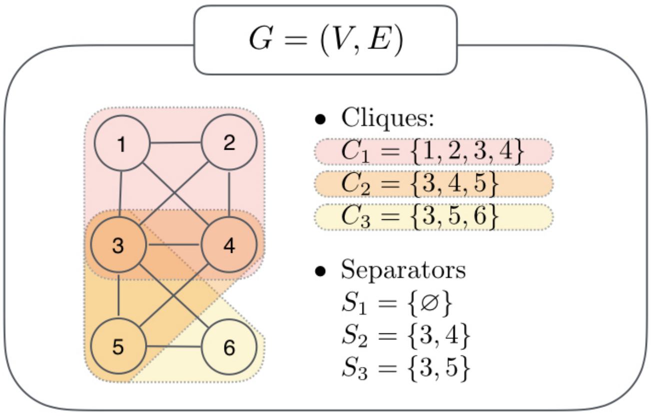

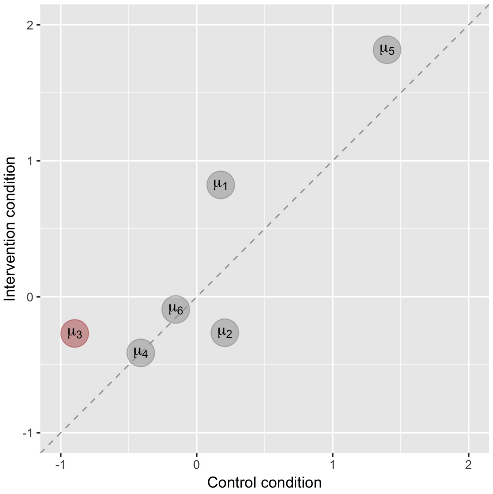

(a) Mean

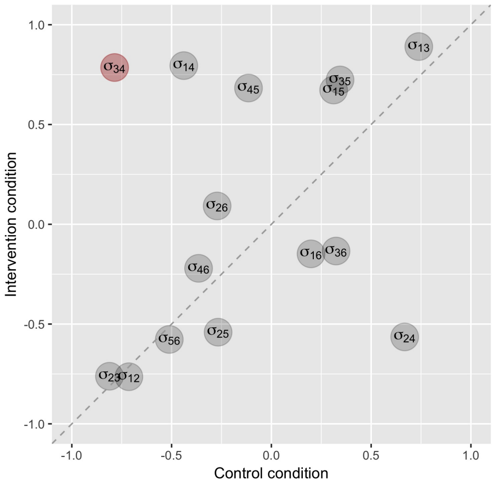

(b) Correlation

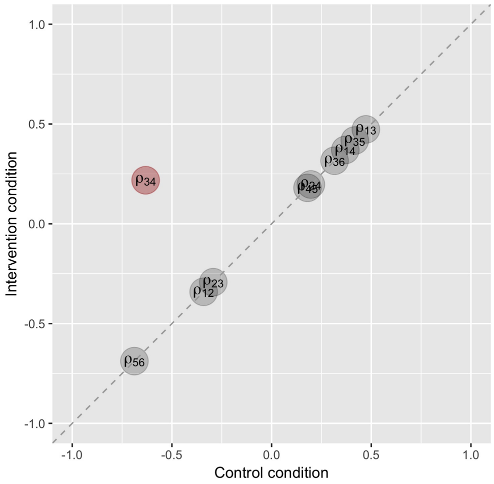

(c) Partial correlation

Figure S2: Mean (a), correlation (b) and partial correlation (c) parameters in the control and the intervention condition. Parameters directly affected by the intervention are highlighted (red). Dashed line $y = x$ added for reference.

Element	A	$\lambda(A)$	df	p-value
C_1	$\{1,2,3,4\}$	231.95	14	1.5×10^{-41}
C_2	$\{3,4,5\}$	225.58	9	1.4×10^{-43}
C_3	$\{3,5,6\}$	48.93	9	1.7×10^{-7}
S_2	$\{3,4\}$	224.71	5	1.4×10^{-46}
S_3	$\{3,5\}$	43.74	5	2.6×10^{-8}

Table S1: Test of equality of distribution of cliques and separators in G. Significant p-values are highlighted.

where $\mu^{(i)}$, $\Sigma^{(i)}$, $i = 1, 2$, are parameters of the control and intervention distribution, respectively. We then consider a decomposition of the global hypothesis induced by the graphical structure of the model. The graph G consists of three cliques: $C_1 = \{1,2,3,4\}$, $C_2 = \{3,4,5\}$, and $C_3 = \{3,5,6\}$. The associated sequence of separators is $S_2 = \{3,4\}$, $S_3 = \{3,5\}$. The perturbation that we have performed affects all these components. As a numerical confirmation, we generated a random sample of size 100 for each condition (i.e., before and after the intervention on X_3 and X_4) and tested equality of the distributions of all cliques and separators (see Table S1). As shown in the table, all grains of the graphical decomposition are marginally statistically different. We therefore exploit multiplicity of the graphical description to try to recover the true source of dysregulation.

There are three possible orderings of these grains satisfying the running intersection property. More formally, let $C_{i,1}, ..., C_{i,3}$ denote the i-th decomposition, $i = 1, \ldots, 3$, having $C_{i,1} = C_i$ as root clique, and $S_{i,1}, ..., S_{i,3}$ be a corresponding sequence of separators, where $S_{i,1} = \varnothing$. This gives rise to the following decompositions:

- **Ordering 1** (root C_1):
  $C_{1,1} = C_1, \quad C_{1,2} = C_2, \quad C_{1,3} = C_3;$
  $S_{1,1} = \varnothing, \quad S_{1,2} = S_2, \quad S_{1,3} = S_3;$
- **Ordering 2** (root C_2):
  $C_{2,1} = C_2, \quad C_{2,2} = C_1, \quad C_{2,3} = C_3;$
  $S_{2,1} = \varnothing, \quad S_{2,2} = S_2, \quad S_{2,3} = S_3;$
- **Ordering 3** (root C_3):
  $C_{3,1} = C_3, \quad C_{3,2} = C_2, \quad C_{3,3} = C_1;$
  $S_{3,1} = \varnothing, \quad S_{3,2} = S_3, \quad S_{3,3} = S_2;$

Correspondingly, the log likelihood ratio (LLR) criterion $\lambda(V)$ for testing H can be decomposed as:

$$\lambda(V) = \lambda(C_1) + [\lambda(C_2) - \lambda(S_2)] + [\lambda(C_3) - \lambda(S_3)]$$
$$\lambda(V) = \lambda(C_2) + [\lambda(C_1) - \lambda(S_2)] + [\lambda(C_3) - \lambda(S_3)]$$
$$\lambda(V) = \lambda(C_3) + [\lambda(C_2) - \lambda(S_3)] + [\lambda(C_1) - \lambda(S_2)]$$

where $\lambda(A)$ denotes the log likelihood ratio criterion associated to the marginal model induced by A. The key observation is that the components in squared brackets on the right-hand sides correspond to tests of equality of conditional distributions. For example, in the first decomposition $[\lambda(C_2) - \lambda(S_2)]$ focuses on the distribution of X_5 given X_3 and X_4, while $[\lambda(C_3) - \lambda(S_3)]$, focuses on the distribution of X_6 given X_3 and X_5. Furthermore, the three components have a limiting chi-squared distribution and are jointly (asymptotically) independent.

Root clique	Component	$\lambda(C_{i,j}) - \lambda(S_{i,j})$	df	p-value	$\hat{D}_{G,i}$
C_1	C_1	231.94	14	1.5×10^{-41}	$\{ C_1 \}$
	$C_2 \vert S_2$	0.87	4	0.93	
	$C_3 \vert S_3$	5.19	4	0.27	
C_2	C_2	225.58	9	1.4×10^{-43}	$\{ C_2 \}$
	$C_1 \vert S_2$	7.23	9	0.61	
	$C_3 \vert S_3$	5.19	4	0.27	
C_3	C_3	48.93	9	1.7×10^{-7}	$\{ C_3 \cup C_2 \}$
	$C_2 \vert S_3$	181.84	4	3.0×10^{-38}	
	$C_1 \vert S_2$	7.23	9	0.61	

Table S2: Marginal and conditional tests for the $k = 3$ decompositions of G. Significant p-values are highlighted.

Let us look at the interpretation of the individual tests. The first decomposition allows us to fill in the first three rows of the (Table S2). The first test is significant at level $\alpha = 0.05$, while the other two are not, although C_2 and C_3 are individually marginally significant (Table S1). This implies that differences in marginal distribution of C_2 and C_3 are fully explained by the changes in the marginal distribution of the first clique.

We now combine results of all three decompositions to estimate the source set. Distinct decompositions of the global null hypothesis give rise to a collection $\{H_{i,j},\ i, j = 1, \ldots, 3\}$ of local hypotheses of equality of the conditional distributions of $X_{C_{i,j} \setminus S_{i,j}} | X_{S_{i,j}},\ i, j = 1, \ldots, 3$, (Table S2). Then if $\phi_{i,j} \in \{0, 1\}$ denotes the outcome of the test of $H_{i,j}$, with $\phi_{i,j} = 1$ when $H_{i,j}$ is rejected, the estimate of the source set is given by

$$\hat{D}_G = \bigcap_{i=1}^{k} \hat{D}_{G,i}$$

where $D_{G,i} = \bigcup_{\{j : d^*_{i,j} = 1\}} C_{i,j}$.

In our example, if we set $\alpha = 0.05$, we obtain $\phi_1 = \{1, 0, 0\}$, $\phi_2 = \{1, 0, 0\}$, and $\phi_3 = \{1, 1, 0\}$, which leads to $\hat{D}_G = \{3, 4\}$, which is indeed the true minimal source set.

References

Allaire, J., C. Gandrud, K. Russell, and C. Yetman (2017). *networkD3: D3 JavaScript Network Graphs from R*. R package version 0.4.

Azzalini, A. (2018). *sn: The Skew-Normal and Related Distributions such as the Skew-t*. R package version 1.5.

Bostock, M., V. Ogievetsky, and J. Heer (2011, December). D3 data-driven documents. *IEEE Transactions on Visualization and Computer Graphics 17*(12), 2301–2309.

Capitanio, A., A. Azzalini, and E. Stanghellini (2003). Graphical models for skew-normal variates. *Scandinavian Journal of Statistics 30*(1), 129–144.

Dethlefsen, C. and S. Hojsgaard (2005). A common platform for graphical models in R: The gRbase package. *Journal of Statistical Software 14*(17), 1–12.

Djordjilović, V., M. Chiogna, and J. Vomlel (2017). An empirical comparison of popular structure learning algorithms with a view to gene network inference. *International Journal of Approximate Reasoning 88*(Supplement C), 602 – 613.

Huang, Y.-T. and X. Lin (2013). Gene set analysis using variance component tests. *BMC Bioinformatics 14*(1), 210.

Lauritzen, S. L. (1996). *Graphical Models*. Oxford University Press.

Sales, G., E. Calura, and C. Romualdi (2017). *graphite: GRAPH Interaction from pathway Topological Environment*. R package version 1.22.0.

Schäfer, J. and K. Strimmer (2005). A shrinkage approach to large-scale covariance matrix estimation and implications for functional genomics. *Statistical Applications in Genetics and Molecular Biology 4*(1).

Wan, Y.-W., G. I. Allen, Y. Baker, E. Yang, P. Ravikumar, and Z. Liu (2015). *XMRF: Markov Random Fields for High-Throughput Genetics Data*. R package version 1.0.

Westfall, P. H. and S. S. Young (1993). *Resampling-based multiple testing: Examples and methods for p-value adjustment*. John Wiley & Sons, New York.
