# Supplementary material for: SourceSet: A graphical model approach to identify primary genes in perturbed biological pathways
Source: PLoS Comput Biol. 2019 Oct 25;15(10):e1007357. doi: 10.1371/journal.pcbi.1007357 (PMC6834292; doi:10.1371/journal.pcbi.1007357)
Supplement: S2 Text — (PDF) [file pcbi.1007357.s002.pdf]

S2 Theoretical foundation

We assume to model the data of the same pathway in two different experimental conditions as realizations of two Gaussian graphical models sharing the same decomposable graph G. Here, $G = (V, E)$ is obtained from the pathway topology conversion, where V and E represent genes and biochemical reactions, respectively. Formally:

$$X^{(i)} \sim \mathcal{N}_p(\mu^{(i)}, \Sigma^{(i)}), \quad (\Sigma^{(i)})^{-1} \in \mathcal{S}^+(G), \qquad i = 1, 2,$$

where p is the number of genes and $\mathcal{S}^+(G)$ is the set of symmetric positive definite matrices with null elements corresponding to the missing edges of G.

A major advantage of decomposable graphs is that they allow for a clique-grained description. Let $C_i,\ i = 1, \ldots, k$ be the cliques of the graph G. These can be arranged so as to satisfy the *running intersection property* Lauritzen (1996). As there are at least k such orderings of the cliques, one for each choice of the root clique, let $C_{i,1}, ..., C_{i,k}$ denote the ith ordering, $i = 1, \ldots, k$, having $C_{i,1} = C_i$ as root clique, and $S_{i,1}, ..., S_{i,k}$ be a corresponding sequence of separators, where $S_{i,1} = \varnothing$. These k orderings provide k alternative descriptions of the same graphical structure.

As graphical models are characterizations of conditional independence structures within distributions, the multiple representations previously introduced translate into alternative factorizations of the joint probability distribution associated to G, and into alternative decompositions of the corresponding inferential problems. Consider the general hypothesis of equality of distribution in the two conditions $H : \Sigma^{(1)} = \Sigma^{(2)}$ and $\mu^{(1)} = \mu^{(2)}$. Given an ordering i of the cliques, $i = 1, \ldots, k$, such global hypothesis decomposes into a set of k independent hypotheses $H_{i,j},\ j = 1, \ldots, k$ of equality of the conditional distributions of $X_{C_{i,j} \setminus S_{i,j}} | X_{S_{i,j}},\ j = 1, \ldots, k$. These can be tested on exploiting an appropriate i-specific decomposition of the log likelihood ratio criterion (LLR):

$$\lambda(V) = \sum_{j=1}^{k} [\lambda(C_{i,j}) - \lambda(S_{i,j})] \tag{1}$$

where the $\lambda(C_{i,j}) - \lambda(S_{i,j})$ terms correspond to the LLR for testing the corresponding $H_{i,j}$.

Since each ordering of the cliques corresponds to an alternative factorization of the same distribution, we exploit this multiplicity to search for the the smallest set, D_G say, respecting the graphical granularity of G, which contains the source set D. To this aim, consider the whole collection of hypothesis $H_{i,j}$ $(i, j = 1, ..., k)$ of equality of distributions of $X_{C_{i,j} \setminus S_{i,j}} | X_{S_{i,j}}$ in two conditions, implied by G.

Proposition 1. *Let $d_i^* = (d_{i,1}^*, ..., d_{i,k}^*)$ be the vector of correct decisions (the truth) for the hypothesis $H_{i,j}$, with $d_{i,j} = 0$ when the hypothesis $H_{i,j}$ is true, and $d_{i,j} = 1$ otherwise. Then D_G, defined as:*

$$D_G = \bigcap_{i=1}^{k} D_{G,i}$$

where $D_{G,i} = \bigcup_{\{j : d_{i,j}^* = 1\}} C_{i,j}$, is a source set.

Proof. By construction, $D \subseteq D_{G,i}\ \forall i$, so that $D \subseteq D_G$. The case $D = D_G$ is trivial. Consider the case $D \subset D_G$. We need to prove that the following two conditions hold for D_G:

1. the distribution of $X_{D_G}^{(1)}$ differs from that of $X_{D_G}^{(2)}$;

2. the conditional distributions $X_{\bar{D}_G}^{(1)} | X_{D_G}^{(1)}$ and $X_{\bar{D}_G}^{(2)} | X_{D_G}^{(2)}$ coincide, where $\bar{D}_G = V \setminus D_G$.

The definition of D implies that the conditional distributions $X^{(1)}_{A\setminus D}|X^{(1)}_D$ and $X^{(2)}_{A\setminus D}|X^{(2)}_D$ coincide for every $A \subseteq V$. Being the distribution of $X^{(j)}_{D_G}$ the product of the distribution of $X^{(j)}_D$ and of $X^{(j)}_{D_G\setminus D}|X^{(j)}_D$, $j = 1,2$, it follows that the distribution of $X^{(1)}_{D_G}$ differs from that of $X^{(2)}_{D_G}$ due to the difference in distribution of $X^{(1)}_D$ and $X^{(2)}_D$. Moreover, the conditional distributions $X^{(j)}_{\bar{D}_G}|X^{(j)}_{D_G}$, $j = 1,2$ can be computed as the ratio of the conditional distributions of $X^{(j)}_{\bar{D}}|X^{(j)}_D$ and of $X^{(j)}_{D_G\setminus D}|X^{(j)}_D$. This give rise, in the two conditions, to ratios having same numerators and denominators, showing that condition 2 is also satisfied. □

Since D_G can be seen as the smallest source set identifiable by means of cliques and separators of the underlying graph, we call it the *graphical source set.* In our setting, the set $\mathbb{D}_{\mathbf{G}} = \bigcup_{i=1}^k D_{G,i}$ contains all genes affected by the perturbation. The set $D_G \subseteq \mathbb{D}_{\mathbf{G}}$ represents the graphical hull of genes which can be deemed to be responsible for the dysregulation. From now on, when no ambiguity can arise, we will refer to D_G simply as the source set (or primary set), and $\mathbb{D}_{\mathbf{G}} \setminus D_G$ as the secondary set.

References

Allaire, J., C. Gandrud, K. Russell, and C. Yetman (2017). *networkD3: D3 JavaScript Network Graphs from R.* R package version 0.4.

Azzalini, A. (2018). *sn: The Skew-Normal and Related Distributions such as the Skew-t.* R package version 1.5.

Bostock, M., V. Ogievetsky, and J. Heer (2011, December). D3 data-driven documents. *IEEE Transactions on Visualization and Computer Graphics 17*(12), 2301–2309.

Capitanio, A., A. Azzalini, and E. Stanghellini (2003). Graphical models for skew-normal variates. *Scandinavian Journal of Statistics 30*(1), 129–144.

Dethlefsen, C. and S. Hojsgaard (2005). A common platform for graphical models in R: The gRbase package. *Journal of Statistical Software 14*(17), 1–12.

Djordjilović, V., M. Chiogna, and J. Vomlel (2017). An empirical comparison of popular structure learning algorithms with a view to gene network inference. *International Journal of Approximate Reasoning 88*(Supplement C), 602 – 613.

Huang, Y.-T. and X. Lin (2013). Gene set analysis using variance component tests. *BMC Bioinformatics 14*(1), 210.

Lauritzen, S. L. (1996). *Graphical Models.* Oxford University Press.

Sales, G., E. Calura, and C. Romualdi (2017). *graphite: GRAPH Interaction from pathway Topological Environment.* R package version 1.22.0.

Schäfer, J. and K. Strimmer (2005). A shrinkage approach to large-scale covariance matrix estimation and implications for functional genomics. *Statistical Applications in Genetics and Molecular Biology 4*(1).

Wan, Y.-W., G. I. Allen, Y. Baker, E. Yang, P. Ravikumar, and Z. Liu (2015). *XMRF: Markov Random Fields for High-Throughput Genetics Data.* R package version 1.0.

Westfall, P. H. and S. S. Young (1993). *Resampling-based multiple testing: Examples and methods for p-value adjustment.* John Wiley & Sons, New York.
