# Supplementary material for: SourceSet: A graphical model approach to identify primary genes in perturbed biological pathways
Source: PLoS Comput Biol. 2019 Oct 25;15(10):e1007357. doi: 10.1371/journal.pcbi.1007357 (PMC6834292; doi:10.1371/journal.pcbi.1007357)
Supplement: S3 Text — (PDF) [file pcbi.1007357.s003.pdf]

S3 Estimation: impact of shrinkage on p-values of LLR tests

The LLR test is well defined whenever the number of samples for the smallest group $n = \min(n_1, n_2)$ is greater than the cardinality of the largest clique $p^* = \max(|C_1|, ..., |C_k|)$, where $|A|$ denotes cardinality of a set A. Indeed, the estimates of the covariance matrices in (2) in the **main paper**, must be positive definite. In practice, high-throughput experiments are usually done with very few replicates due to budgetary constraints, which makes the proposed method applicable to a limited number of cases (see Table 1 in the main paper). Moreover, even when the number of samples is sufficient (i.e., $n \approx p^*$) and the maximum likelihood estimate exists, the sample covariance matrix can no longer be considered a good estimate of the covariance matrix.

Great efforts have been undertaken to gain efficiency in large-scale covariance estimation with small-sample data. Among the available strategies, shrinkage methods appear to be a valuable option. See, for example, Schäfer and Strimmer (2005), which is shown to enjoy certain optimality properties within the "large p, large n" asymptotics.

In the source set setting, i.e., two-sample comparison, different shrinking strategies could be employed. Consider, for example, the problem of selecting the tuning parameter that controls the amount of shrinkage during estimation, a parameter whose asymptotically optimal value for a given matrix is derived in Schäfer and Strimmer (2005) . The simplest approach would be to take the same tuning parameter for estimating the three covariance matrices needed in the procedure, i.e., the covariance matrices in the first and second condition and the pooled covariance. In this case, the question comes naturally as to which of the three possible optimal tuning parameters should be preferred. Alternatively, the optimal tuning parameter could be employed for each matrix. To the best of our knowledge, a discussion of the best shrinking strategy and of its impact on the validity of p-values in the context of two-sample testing procedure is not available in the literature.

To fill in this gap, we performed a simulation study aimed at verifying if, under the null hypothesis of no dysregulation between two conditions, three alternative shrinking strategies based on Schäfer and Strimmer (2005), named *min*, *max*, *optimal*, (see Section S3.1 for details) affect the theoretical distribution of the p-values of LLR tests used in source set, which is known to be uniform. This is important to avoid systematic bias in the statistical testing procedures on which the source set procedure is based.

We derived Monte Carlo estimates of the distribution of p-values from LLR tests for two simulation scenarios built around a complete graph on a set C of $p = 10$ variables and $n_1 = n_2 = 10$ statistical units. In Scenario a, absence of dysregulation was simulated; in Scenario b, a subset $S \subset C$ of variables was perturbed so as to give rise to distributions for X_C and X_S different in the two conditions, while preserving the same distribution for $X_{C \setminus S} \mid X_S$. Three null hypotheses were tested in both scenarios, namely, equality of the distributions of X_C, of X_S and of $X_{C \setminus S} \mid X_S$, using LLR tests introduced in Section **Estimation** of the main paper. In the second scenario, therefore, only the third hypothesis is true.

In Figure S3, we show example violin plots of the distribution of p-values in the two scenarios for the three hypotheses and the three shrinking strategies. Inspection of the plots reveals that, in Scenario a, the distributions of p-values fit the theoretical distribution very well, whereas, in Scenario b the distribution of p-values for the hypothesis of equality of the distribution of $X_{C \setminus S} \mid X_S$ does not fit to the theoretical one for all shrinking strategies, implying that shrinkage is likely to bias the source set procedure.

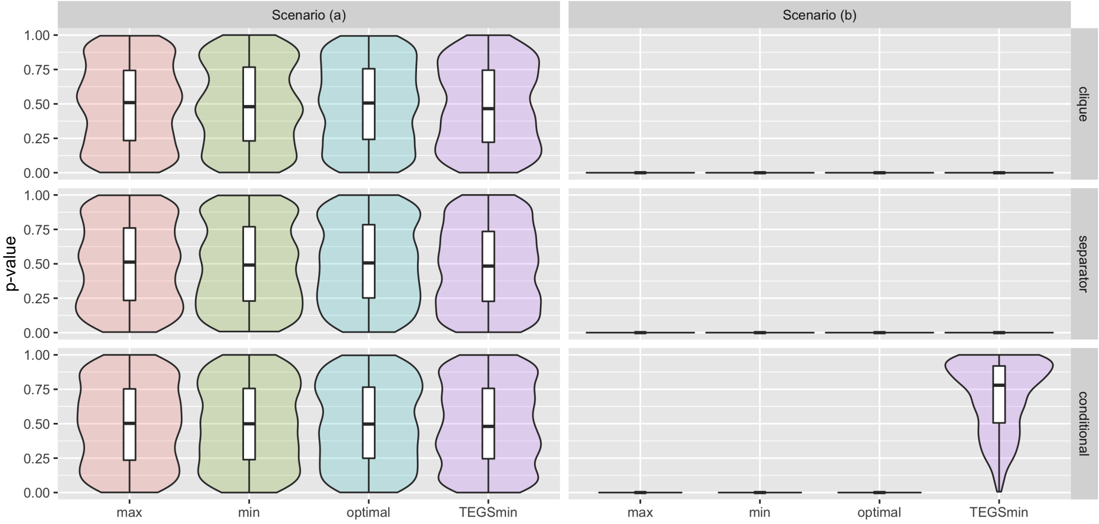

Figure S3: Distributions (500 Monte Carlo runs) of the p-values under Scenario a (left panel) and Scenario b (right panel) for testing equality of distribution of X_C (clique $C = \{1, \cdots, 10\}$), X_S (separator $S = \{6, ..., 10\}$), and $X_{C\setminus S} \mid X_S$ (conditional) in two conditions ($n_1 = n_2 = 10$).

For this reason, we introduce a new estimation strategy, named *TEGSmin*, based on an *ad-hoc* ridge estimator Huang and Lin (2013), that adds a small quantity to the diagonals of the three covariance matrices.To determine this quantity, we: i) find the distribution of the sample variances of the p variables in the two groups and in the pooled sample; ii) compute the fifth percentile of each of these distributions iii) use the minimum as the tuning parameter. This procedure allows to stabilize the estimates of the covariance matrices, while maintaining the validity of the testing procedures. This is evidenced by the distribution of the above-mentioned p-value in Figure S3 (bottom right panel), which is stochastically larger than the uniform, meaning that we obtained a valid, although conservative, testing procedure. Such findings have also been confirmed with different configurations of sample sizes (n_1 and n_2), number p of variables considered and of dysregulation regimes (results not shown here).

S3.1 Details of simulations

Given a fully connected graph on a set C of 10 genes, we have simulated $n_1 = 10$ observations for a fixed mean and a fixed covariance matrix representing the control condition. We then considered two possibilities for the second condition:

(*Scenario* a) $n_2 = 10$ additional observations were simulated from the distribution of the control condition;

(*Scenario* b) $n_2 = 10$ observations were simulated from a distribution in which a subset S of 6 genes was perturbed, while the conditional distribution of the remaining genes given S was held fixed. In this way, we ensured that the distributions of X_C and X_S are different in two conditions, while the conditional distribution of $X_{C\setminus S} \mid X_S$ remains the same.

The above procedure was repeated $B = 500$ times, so that for each Monte Carlo run, we obtained two datasets of 10 observations of 10 genes. Let $S^{(1)}$, $S^{(2)}$, and S^{pooled} represent

sample covariance matrices, computed in each run, for condition 1, condition 2, and for the pooled sample, respectively.

We then considered three different shrinking strategies. In particular, Schäfer and Strimmer (2005) shrink the sample correlation matrix towards an identity matrix and the vector of sample variances towards its median, where the amount of shrinkage is determined by two shrinkage parameters λ and λ_{var}. Schäfer and Strimmer (2005) derive an asymptotically optimal amount of shrinkage for a given sample covariance matrix. In what follows, we discuss choices for λ, but the analogous considerations apply to λ_{var}.

Let us denote by λ_1, λ_2, λ_{pooled} the optimal shrinkage parameters for the correlation matrices associated to $S^{(1)}$, $S^{(2)}$ and S^{pooled}. We considered the following choices:

- **max**: $\lambda_{max} = \max\{\lambda_1, \lambda_2, \lambda_{pooled}\}$;
- **min**: $\lambda_{min} = \min\{\lambda_1, \lambda_2, \lambda_{pooled}\}$;
- **optimal**: each sample covariance matrix is shrank with its own optimal λ.

With each of the three above given choices of λ, we have computed regularized estimates of the covariance matrices of the two conditions, as well as of the common covariance matrix under the null hypothesis. This allowed us to compute LLR for C and S, as shown in (4) (main text). Given the small sample size, to obtain p-values for the tests of equality of X_C, X_S and $X_{C\setminus S} \mid X_S$ in two conditions, we relied on permutations. We performed this procedure for each Monte Carlo run which gave us a sample from the distribution of p-values for each of the three tests, and for each of the three shrinkage choices.

All the functions used to derive the optimal parameters and the shrank covariance matrices can be found in the `corpcor` package, through the `cov.shrink, estimate.lambda, estimate.lambda.var` functions.

References

Allaire, J., C. Gandrud, K. Russell, and C. Yetman (2017). *networkD3: D3 JavaScript Network Graphs from R.* R package version 0.4.

Azzalini, A. (2018). *sn: The Skew-Normal and Related Distributions such as the Skew-t.* R package version 1.5.

Bostock, M., V. Ogievetsky, and J. Heer (2011, December). D3 data-driven documents. *IEEE Transactions on Visualization and Computer Graphics 17*(12), 2301–2309.

Capitanio, A., A. Azzalini, and E. Stanghellini (2003). Graphical models for skew-normal variates. *Scandinavian Journal of Statistics 30*(1), 129–144.

Dethlefsen, C. and S. Hojsgaard (2005). A common platform for graphical models in R: The gRbase package. *Journal of Statistical Software 14*(17), 1–12.

Djordjilović, V., M. Chiogna, and J. Vomlel (2017). An empirical comparison of popular structure learning algorithms with a view to gene network inference. *International Journal of Approximate Reasoning 88*(Supplement C), 602 – 613.

Huang, Y.-T. and X. Lin (2013). Gene set analysis using variance component tests. *BMC Bioinformatics 14*(1), 210.

Lauritzen, S. L. (1996). *Graphical Models.* Oxford University Press.

Sales, G., E. Calura, and C. Romualdi (2017). *graphite: GRAPH Interaction from pathway Topological Environment.* R package version 1.22.0.

Schäfer, J. and K. Strimmer (2005). A shrinkage approach to large-scale covariance matrix estimation and implications for functional genomics. *Statistical Applications in Genetics and Molecular Biology 4*(1).

Wan, Y.-W., G. I. Allen, Y. Baker, E. Yang, P. Ravikumar, and Z. Liu (2015). *XMRF: Markov Random Fields for High-Throughput Genetics Data.* R package version 1.0.

Westfall, P. H. and S. S. Young (1993). *Resampling-based multiple testing: Examples and methods for p-value adjustment.* John Wiley & Sons, New York.
