# Supplementary material for: SourceSet: A graphical model approach to identify primary genes in perturbed biological pathways
Source: PLoS Comput Biol. 2019 Oct 25;15(10):e1007357. doi: 10.1371/journal.pcbi.1007357 (PMC6834292; doi:10.1371/journal.pcbi.1007357)
Supplement: S4 Text — (PDF) [file pcbi.1007357.s004.pdf]

S4 Multiple testing correction

Estimation of D_G requires performing a collection of $k+\sum_{i=1}^{k}\nu(C_i)$ tests, where k is the number of cliques in G and $\nu(C_i)$ denotes the number of separators contained within the clique C_i. Finite sample behavior of $\hat{D}_G$ thus hinges on the proper control of the multiplicity issue. If we wish to control the inclusion of false positives in $\hat{D}_G$ by controlling the familywise error rate (FWER), the simplest approach would be to apply the Bonferroni correction with a factor of $k+\sum_{i=1}^{k}\nu(C_i)$. However, the Bonferroni correction can be overly conservative in this situation since intricate logical relations among subsets of hypotheses result in a high positive dependence between the associated p-values.

To address this problem we use a method proposed by Westfall and Young Westfall and Young (1993), which uses permutations to obtain the joint distribution of the p-values and, by considering their structure of dependence, improves the power with respect to the Bonferroni procedure.

The procedure proposed by these authors, also called max$T$, starts with the creation of T permuted datasets and calculates the m test statistics for each of these. The results can be arranged in a $(T+1)\times m$ matrix P, where the first row is filled with the statistics calculated on the original data, while the remaining T store the test statistics of the permuted datasets. For a given level α, we proceed as follows:

step 1) for each of the m columns of P, we calculate the asymptotic p-values;

step 2) for each of the $(T+1)$ rows of P, we calculate the minimum p-value;

step 3) the corrected threshold θ is the α-quantile of the permutational distribution of the p-values obtained in the previous step.

In order to increase the power, we decided to use a *step-down* version of the algorithm. Then, at the end of the three steps for the first iteration, we remove from the matrix P all the columns associated to rejected tests using the corrected threshold θ. Steps (2) and (3) are repeated on the resized P matrix until no hypothesis is rejected, considering the θ_i threshold for the i-th iteration.

In the guided example, the number of nodes was quite small ($|V|=6$) with respect to the sample size ($n=100$). In practice, the situation is often the opposite: the number of genes greatly prevails over the number of statistical units. In that case, the likelihood ratio criterion cannot be used and we need to resort to some regularization procedures. If a ridge estimate of the covariance matrix is used, as we propose in our approach, the asymptotic distribution is no longer valid, and we have to rely on permutations to obtain p-values. In this case, we use the min$P$ method, and compute permutational p-values per-hypothesis to obtain the $\widetilde{P}$ matrix, where each $\widetilde{p}_{ij}$ is defined as $\#\{l : p_{lj}\leq p_{ij}\}$. The matrix $\widetilde{P}$ replaces the P matrix in the max$T$ algorithm.
