# Supplementary material for: SourceSet: A graphical model approach to identify primary genes in perturbed biological pathways
Source: PLoS Comput Biol. 2019 Oct 25;15(10):e1007357. doi: 10.1371/journal.pcbi.1007357 (PMC6834292; doi:10.1371/journal.pcbi.1007357)
Supplement: S5 Text — (PDF) [file pcbi.1007357.s005.pdf]

S5 Algorithm

Looking at the broad picture of the N pathways case, to make the procedure coherent some important practical issues have to be addressed.

S5.1 (*Note 1*) List of pathways

The interest of our work is not in the detection of the structure of a pathway because we consider it fixed a priori. Various research groups have tried different strategies to address this challenge that have led to the development of many knowledge databases. To incorporate pathways into graphical models, the diagram needs to be translated into a directed or undirected graph. Due to the descriptive nature of the pathways and their inherent complexity, there is no simple recipe for conversion that can be applied in every situation. For this reason, close collaboration with biologists is preferred at this step Djordjilović et al. (2017).

In general, we give full freedom to the user in providing the underlying graph, requiring only a specific input format (i.e., a `graphNEL` object). So, the user can provide a list of manually curated pathways or use developed softwares to translate the bases of knowledge. To date, the most complete software available for this task is `graphite` R package Sales et al. (2017). `graphite` provides easy access to six different databases for a total of 14 different species. The resulting networks represent a standardized resource for the pathway analysis.

Regardless of the type of graph, obtained at the end of the translation (i.e., undirected or directed), our method works only with decomposable undirected structures. However, it should be stressed that we can always obtain a decomposable graph in few steps (i.e., moralization and triangulation).

S5.2 (*Note 2*) Setting the parameters

The input pathways normally have heterogeneous sizes and degrees of connectivity. To make the results obtained from different graphs comparable, and to conduct a meta-analysis, particular attention is needed with respect to the main options regarding:

- estimation of the covariance matrices, i.e., the maximum likelihood estimation or the regularized estimation;
- the number of permutations for the min$P$ or max$T$ procedure.

The estimation method must be the same for all pathways. If the user wants to use the maximum likelihood estimation, all cliques in all pathways must satisfy the $n > p_i$ condition, where n is the number of samples for the smaller class and p_i the cardinality of the largest clique in the i-th pathway. If even one clique does not satisfy this requirement, the regularized estimate will be used. To obtain reliable results for the maximum likelihood case, it is recommended to use as criterion $n \gg p_i$. Indeed, the distribution used to calculate the p-values of the performed tests is only asymptotically valid.

The number of permutations T_i, whether it is the max$T$ or min$P$ correction, is naturally suggested from the α threshold and the number m_i of unique tests in the i-th pathway (see Section S4). Using different thresholds allows to control the FWER for each pathway, and achieve comparable power among pathways.

It is worth nothing that the FWER can also be controlled simultaneously across all pathways; it would be sufficient to use a single $(T_M + 1) \times M$ matrix P, where M is the number of unique tests performed in all pathways, that is $M = \sum_{i=1}^{N} m_i$. The main problem is that the number

T_M is generally very large, making the algorithm computationally onerous. Besides, the results may lose reproducibility as the results for a given pathway would depend on the number and the degree of connectivity of the other input graphs. For these reasons, this option is not considered in the implemented algorithm.

S5.3 (*Step 1-2*) Decomposition and orderings

For each pathway and corresponding graph G, the first step requires identifying the maximal cliques and all possible decompositions of the global distribution induced by G. Generally speaking, the *clique problem* is NP-complete, indeed it is fixed-parameter intractable and hard to approximate. Listing maximal cliques can take an exponential time. Therefore, much of the theory about the clique problem is devoted to identifying appropriate types of graphs that admit more efficient algorithms. In our model, a considerable computational relief is possible because decomposable graphs – also called chordal graphs – fall into this category. Also, the detection of permissible decompositions is closely related to the identification of perfect orderings, and such a problem may be solved in polynomial time when the input is a chordal graph.

Our algorithm uses the `rip` function implemented in the `gRbase` package Dethlefsen and Hojsgaard (2005). This function identifies a sequence of cliques that satisfies the running intersection property by first ordering nodes by the maximum cardinality search algorithm. The `root` argument is used to control which clique will first enter the rip ordering.

In the `ripAllRootsClique` function (implemented in the `SourceSet`) we extended the search space to get all possible orderings leading to distinct decompositions, that is, using as root all maximal cliques induced by the graph G. Given a graph, the function will provide:

- a list of k maximal cliques, the associated k separators, and m unique components;
- a list of k orderings; each of them will contain a proper subset of size k of the m unique components.

References

Allaire, J., C. Gandrud, K. Russell, and C. Yetman (2017). *networkD3: D3 JavaScript Network Graphs from R.* R package version 0.4.

Azzalini, A. (2018). *sn: The Skew-Normal and Related Distributions such as the Skew-t.* R package version 1.5.

Bostock, M., V. Ogievetsky, and J. Heer (2011, December). D3 data-driven documents. *IEEE Transactions on Visualization and Computer Graphics 17*(12), 2301–2309.

Capitanio, A., A. Azzalini, and E. Stanghellini (2003). Graphical models for skew-normal variates. *Scandinavian Journal of Statistics 30*(1), 129–144.

Dethlefsen, C. and S. Hojsgaard (2005). A common platform for graphical models in R: The gRbase package. *Journal of Statistical Software 14*(17), 1–12.

Djordjilović, V., M. Chiogna, and J. Vomlel (2017). An empirical comparison of popular structure learning algorithms with a view to gene network inference. *International Journal of Approximate Reasoning 88*(Supplement C), 602 – 613.

Huang, Y.-T. and X. Lin (2013). Gene set analysis using variance component tests. *BMC Bioinformatics 14*(1), 210.

Lauritzen, S. L. (1996). *Graphical Models.* Oxford University Press.

Sales, G., E. Calura, and C. Romualdi (2017). *graphite: GRAPH Interaction from pathway Topological Environment.* R package version 1.22.0.

Schäfer, J. and K. Strimmer (2005). A shrinkage approach to large-scale covariance matrix estimation and implications for functional genomics. *Statistical Applications in Genetics and Molecular Biology 4*(1).

Wan, Y.-W., G. I. Allen, Y. Baker, E. Yang, P. Ravikumar, and Z. Liu (2015). *XMRF: Markov Random Fields for High-Throughput Genetics Data.* R package version 1.0.

Westfall, P. H. and S. S. Young (1993). *Resampling-based multiple testing: Examples and methods for p-value adjustment.* John Wiley & Sons, New York.
