# Supplementary material for: SourceSet: A graphical model approach to identify primary genes in perturbed biological pathways
Source: PLoS Comput Biol. 2019 Oct 25;15(10):e1007357. doi: 10.1371/journal.pcbi.1007357 (PMC6834292; doi:10.1371/journal.pcbi.1007357)
Supplement: S6 Text — (PDF) [file pcbi.1007357.s006.pdf]

S6 Simulation studies under the violation of the symmetry assumption

To asses the performance under the violation of the symmetry assumption, we generated data from two skew-normal multivariate distributions sharing the same decomposable graph G (Fig 2, main text). In particular, for the two conditions we used the previous means and covariance matrices, and a vector of additional skewness parameters.

To obtain realistic results, we used the log count data of `brcadat` dataset (`XMRF` package Wan et al. (2015)) to estimate:

- the expected number of skew-normal marginal univariate distributions by estimating the proportion of genes for which the assumption of symmetry is rejected through Monte Carlo procedure (null hypothesis: skewness parameter is zero).
- the skewness parameter that regulates the asymmetry.

Distributions of the log-count for the two genes in the `brcadat` dataset are shown in Figure S4. Since about 56% of the univariate distributions resulted symmetric (FWER controlled at level $\alpha = 0.01$), we added skewness to 4 out 10 variables. In particular, to preserve the pairwise conditional independences encoded in G (for more details see Proposition 2 in Capitanio et al. (2003)), we selected the variables $\{5,8,9,10\}$.

All the functions used to estimate the skewness parameters and generate observations from the random multivariate skew-normal distribution can be found in the `sn` package Azzalini (2018), through the `rmsn, sn.mple, cp2dp` functions.

The results are reported in Fig.S6.

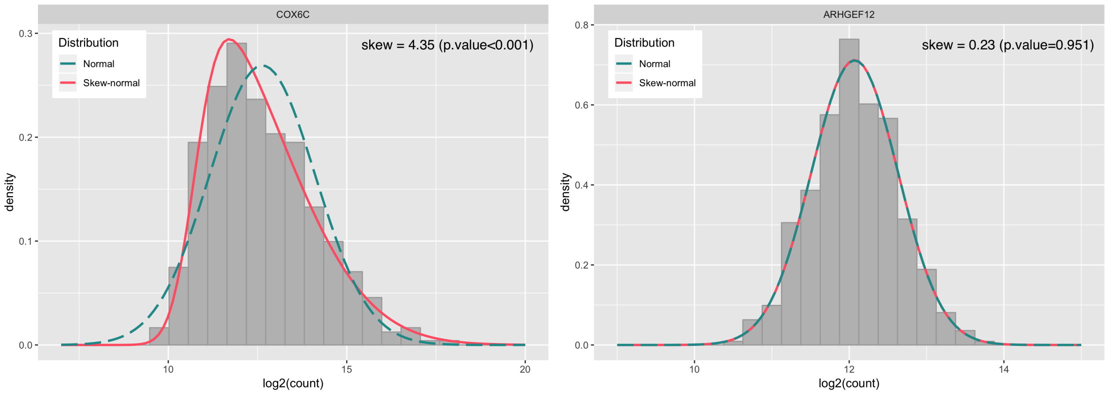

Figure S4: Distribution of the log-counts of two selected genes in the `brcdat` dataset. Density under the null hypothesis that the skew parameter is zero is plotted in green (dashed line); density under the alternative hypothesis is plotted in red. Reported p-values for the hypothesis that the skewness parameter is zero.
