# Supplementary material for: SourceSet: A graphical model approach to identify primary genes in perturbed biological pathways
Source: PLoS Comput Biol. 2019 Oct 25;15(10):e1007357. doi: 10.1371/journal.pcbi.1007357 (PMC6834292; doi:10.1371/journal.pcbi.1007357)
Supplement: S7 Text — (PDF) [file pcbi.1007357.s007.pdf]

S7 SourceSet R package functions

The `SourceSet` package consists of four main functions: the (`sourceSet`) implements the algorithm described in the previous section, while the remaining three functions (`infoSource, easyLookSource, sourceSankeyDiagram`) guide the user in interpreting the obtained results by providing additional statistics and graphical devices. The following sections describe the arguments required by each function, and the provided outputs.

S7.1 Main function

Let us start exploring the package through the `sourceSet` main function. The function necessarily requires the following arguments:

`graphs`	a list of `graphNEL` objects representing the pathways to be analyzed;
`data`	a matrix of expression levels with column names for genes and row names for samples;
`classes`	a vector of length equal to the number of rows of `data`. It indicates the class (condition) of each statistical unit. Only two classes, labeled as 1 and 2, are allowed;
`alpha`	the significance level for FWER control;
`shrink`	if `TRUE`, regularized estimation of the covariance matrices is performed; otherwise, maximum likelihood estimates are used;
`permute`	if `TRUE` permutation p-values are provided; if `FALSE`, asymptotic p-values are returned.

A control is performed on the number of samples in `data` provided. If needed, `shrink` and `permute` are modified and set to appropriate values through internal controls.

A progress bar will show, for each pathway, the status of computations and the elapsed time. The output of the main function is an object of the `sourceSetList` class. It contains as many lists as the input graphs, and provides the following information:

`primarySet`	a character vector containing the names of the variables belonging to the estimated source set $\hat{D}_G$ (primary dysregulation);
`secondarySet`	a character vector containing the names of the variables belonging to $\hat{\mathbb{D}}_{\mathbf{G}} \setminus \hat{D}_G$ (secondary dysregulation);
`orderingSet`	a list of character vectors containing the names of the variables belonging to the estimated source set of each ordering, i.e., $\hat{D}_{G,i}$, $i = 1, \ldots, k$. The union of these elements contains all genes affected by some form of perturbation, i.e., $\hat{\mathbb{D}}_{\mathbf{G}}$;
`Components`	a data frame that contains the information about the m unique tests of the form $C_i \setminus S_i \mid S_i$, including the associated p-values;
`Decompositions`	a list of data frames, one for each identified ordering. Each data frame is a subset of size k (i.e., number of cliques), of the `Components` elements.

`Elements`	cliques and separators of the underlying decomposable graph (see `Graph`)
`Threshold`	a list with information regarding the multiple testing correction: • `alpha`: the input (nominal) significance level; • `value`: the corrected threshold that ensures the control of FWER at level `alpha`; • `type`: the used procedure (min$P$ or max$T$); • `iterations`: the number of iterations for the *step-down* procedure; • `nperms`: the number of permutations.
`Graph`	decomposable graph used in the analysis. It may differ from the input graph. In fact, if the input graph is not decomposable, the function will internally moralize and triangulate it.

S7.2 Pooling results from single-pathway analyses and visualization

Although the interpretation of the source set for a single graph is intuitive, the interpretation of the results obtained from N pathways might be complex. For this reason, we propose a guideline for the meta-analysis providing descriptive statistics and predefined plots. The key input argument of the meta-analysis functions is an object of `sourceSetList` class, that is the output of the `sourceSet` function. Additional arguments may be needed to customize the display.

S7.2.1 `infoSource`

The `infoSource` provides a summary of the results by focusing on either nodes or pathways; in fact, it supplies two different lists that are composed as follows:

	`$graph`
`n.primary`	number of genes belonging to the source set, i.e, $|\hat{D}_G|$ (primary dysregulation) ;
`n.secondary`	number of genes belonging to the secondary set $|\hat{\mathbb{D}}_{\mathbf{G}} \setminus \hat{D}_G|$ (secondary dysregulation);
`n.graph`	number of genes in the graph, i.e., $|V|$;
`n.cluster`	number of connected components in G ;
`primary.impact`	relative size of the estimated source set. This index quantifies the proportion of the graph impacted by the primary dysregulation;
`total.impact`	relative size of the set of genes impacted by dysregulation. This index quantifies the proportion of the graph impacted by either the primary or the secondary dysregulation;
`p.value`	multiplicity adjusted p-value for the hypothesis of equality of the two global distributions associated to the given graph.

	$variable
`n.graph`	number of pathways in which the gene is annotated;
`specificity`	percentage of input graphs containing the given variable with respect to the total number of input graphs;
`primary.impact`	percentage of input graphs, such that the given gene belongs to their estimated source set, with respect to the total number of input graphs in which the gene appears;
`total.impact`	percentage of input graphs, such that the given gene is affected by some form of dysregulation in the considered graph, with respect to the total number of input graphs in which the gene appears;
`relevance`	percentage of the input graphs such that the given variable belongs to their estimated source set, with respect to the total number of input graphs. It is a general measure of the importance of the gene relative to the chosen pathways;
`score`	a number ranging from 0 (low significance) to $+\infty$ (maximal significance), computed as the combination of the p-values of all components (of all the input graphs) containing the given variable. Formally, it is defined as $-\log(\sum_{p=1}^{P} score_p^x/P)$ for $p = 1,..,P$, where P is the number of pathways and x is the considered gene. We define, $score_p^x = \max(\widetilde{p}_{i,j}^x)$ for $i = 1,..k_p$, where $\widetilde{p}_i^x$ is the rescaled p-value using the corrected threshold that ensure the control of FWER (i.e, `value`), for the j-th component in the i-th ordering, such that $X_{\{C_{i,j}/S_{i,j}\}}$ contains x.

S7.2.2 `easyLookSource`

The function `easyLookSource` allows to summarize the results of the analysis through a heatmap (Figure S5b). The plot is composed of a matrix whose rows represent pathways and columns represent genes.

Each cell i, j can take one of the following configurations:

(2) *blue* color, if the i-th gene is in the primary set of the j-th pathway, i.e., $\hat{D}_G$;

(1) *light blue* color, if the i-th gene is in the secondary set of the j-th pathway, i.e., $\hat{\mathbb{D}}_{\mathbf{G}} \setminus \hat{D}_G$;

(0) *gray*, if the i-th gene belongs to the j-th pathway;

(NA) *white*, if the i-th gene does not belong to the j-th pathway.

In the plot, the pathways are vertically ordered from top to bottom according to the numbers of nodes in the source set. Instead, genes are horizontally ordered from left to right based on the number of times they appear in a source set.

S7.2.3 `sourceSankeyDiagram`

Another way to visualize the results is through a Sankey diagram (Figure S5c). It highlights the relationships among nodes, graphs, and source sets.

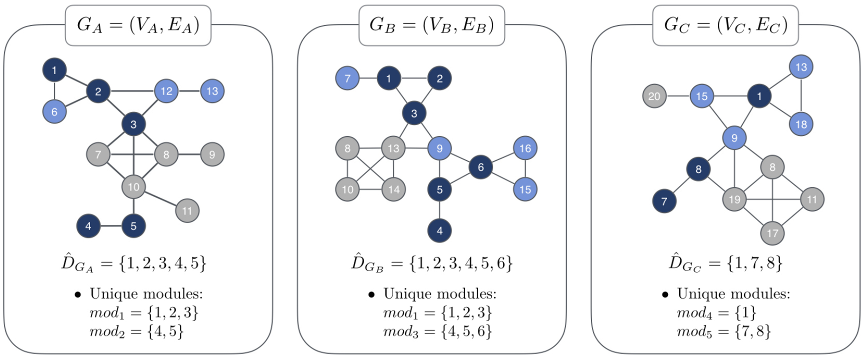

(a) Unique modules

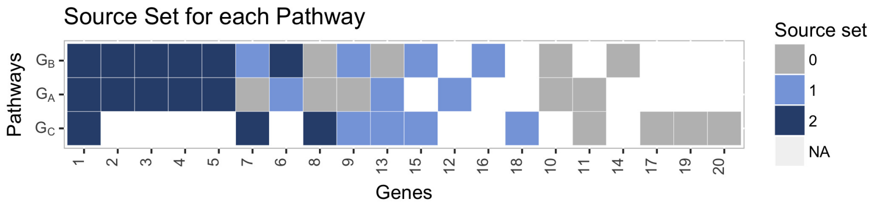

(b) `easyLookSource` function

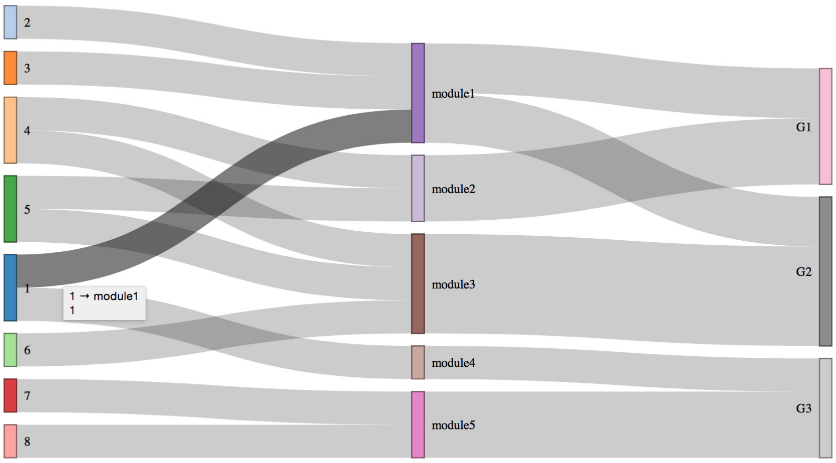

(c) `sourceSankeyDiagram` function

Figure S5: Visualization of the results obtained from the `sourceSet` analysis on an example database consisting of three pathways and twenty genes. For each graph, the modules are identified on the basis of the source set estimate (Figure S5a). The unique modules are used in the representation of the interactive Sankey graph (Figure S5c). The `easylookSource` graph is depicted in Figure S5b. The heat map highlights whether a gene belongs to the null, to the primary, or to the secondary set of a given pathway. For example, gene *7* is in the primary set of the G_C graph (blue rectangle) and in the secondary set of the G_A graph (light blue rectangle). Gene *10* is contained in both the G_A and G_B graphs, but is never be affected by any form of dysregulation (gray rectangles); while it does not appear in the G_C graph (white rectangle).

The layout is organized on three levels:

- the first level (left) shows nodes that appear in at least one of the N source sets.
- the second level (center) is made up of modules (Figure S5a). A module is defined as a set of nodes belonging to a connected subgraph of one pathway, that is also contained in the associated source set. A pathway can have multiple modules, and, at the same time, one module can be contained in multiple pathways.
- the third level (right) shows pathways.

The three levels are to be read from left to right. A link between a left element a and a right element b is to be interpreted as $a \subseteq b$.

The implementation of the `sourceSankeyDiagram` function takes advantage of the D3 library Bostock et al. (2011); Allaire et al. (2017) (JavaScript), making the plot interactive. In fact, it is possible to vertically shift the displayed elements, and to view some useful information by positioning the cursor over items and links.

References

Allaire, J., C. Gandrud, K. Russell, and C. Yetman (2017). *networkD3: D3 JavaScript Network Graphs from R.* R package version 0.4.

Azzalini, A. (2018). *sn: The Skew-Normal and Related Distributions such as the Skew-t.* R package version 1.5.

Bostock, M., V. Ogievetsky, and J. Heer (2011, December). D3 data-driven documents. *IEEE Transactions on Visualization and Computer Graphics 17*(12), 2301–2309.

Capitanio, A., A. Azzalini, and E. Stanghellini (2003). Graphical models for skew-normal variates. *Scandinavian Journal of Statistics 30*(1), 129–144.

Dethlefsen, C. and S. Hojsgaard (2005). A common platform for graphical models in R: The gRbase package. *Journal of Statistical Software 14*(17), 1–12.

Djordjilović, V., M. Chiogna, and J. Vomlel (2017). An empirical comparison of popular structure learning algorithms with a view to gene network inference. *International Journal of Approximate Reasoning 88*(Supplement C), 602 – 613.

Huang, Y.-T. and X. Lin (2013). Gene set analysis using variance component tests. *BMC Bioinformatics 14*(1), 210.

Lauritzen, S. L. (1996). *Graphical Models.* Oxford University Press.

Sales, G., E. Calura, and C. Romualdi (2017). *graphite: GRAPH Interaction from pathway Topological Environment.* R package version 1.22.0.

Schäfer, J. and K. Strimmer (2005). A shrinkage approach to large-scale covariance matrix estimation and implications for functional genomics. *Statistical Applications in Genetics and Molecular Biology 4*(1).

Wan, Y.-W., G. I. Allen, Y. Baker, E. Yang, P. Ravikumar, and Z. Liu (2015). *XMRF: Markov Random Fields for High-Throughput Genetics Data.* R package version 1.0.

Westfall, P. H. and S. S. Young (1993). *Resampling-based multiple testing: Examples and methods for p-value adjustment.* John Wiley & Sons, New York.
