# Supplementary material for: SourceSet: A graphical model approach to identify primary genes in perturbed biological pathways
Source: PLoS Comput Biol. 2019 Oct 25;15(10):e1007357. doi: 10.1371/journal.pcbi.1007357 (PMC6834292; doi:10.1371/journal.pcbi.1007357)
Supplement: S1 Fig — On the left, results based on the maximum likelihood estimate of the covariance matrix; on the right results based on the regularized estimate. Each subpanel corresponds to a different combination of the sample size (columns) and the intensity of dysregulation (rows). Inside subpanels, for each variable Xv, v ∈ V a stacked bar chart shows the percentages of times that v∈D^G (red, primary set), v∈D^G\D^G (orange, secondary set) and v∈V\D^G (green). (PDF) [file pcbi.1007357.s008.pdf]

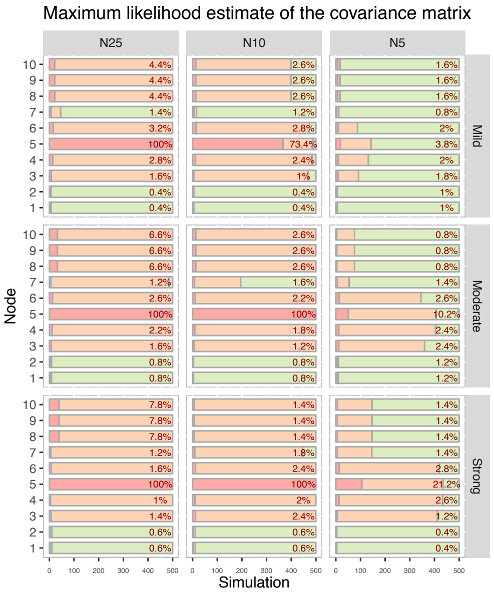
Maximum likelihood estimate of the covariance matrix
N25
N10
N5
Mild
Moderate
Strong
Node
Simulation
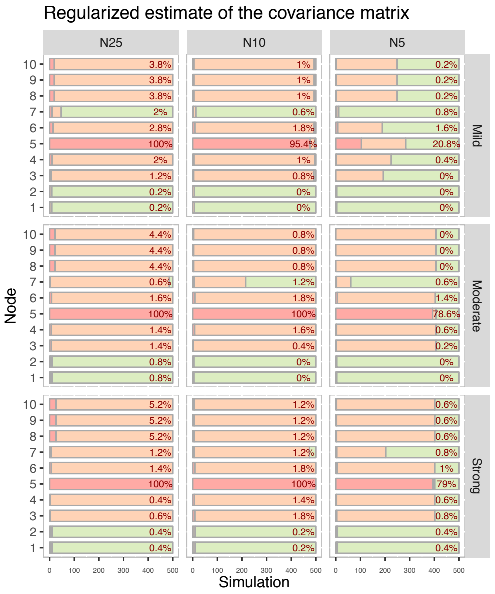
Regularized estimate of the covariance matrix
N25
N10
N5
Mild
Moderate
Strong
Node
Simulation
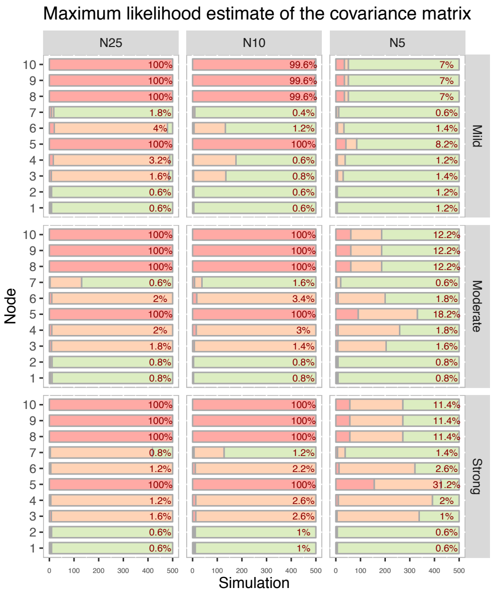
Maximum likelihood estimate of the covariance matrix
N25
N10
N5
Mild
Moderate
Strong
Node
Simulation
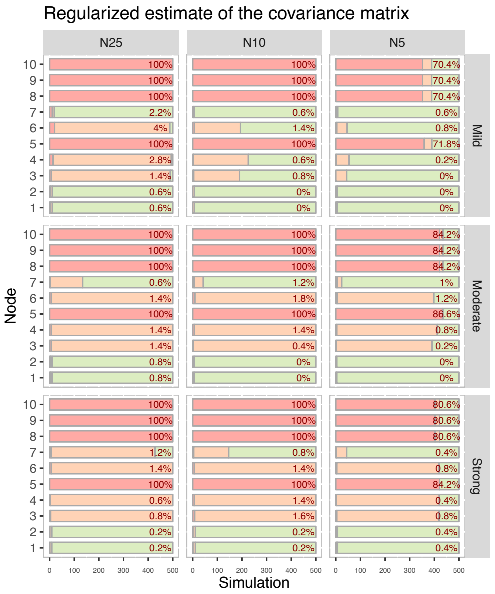
Regularized estimate of the covariance matrix
N25
N10
N5
Mild
Moderate
Strong
Node
Simulation
