# Supplementary material for: SourceSet: A graphical model approach to identify primary genes in perturbed biological pathways
Source: PLoS Comput Biol. 2019 Oct 25;15(10):e1007357. doi: 10.1371/journal.pcbi.1007357 (PMC6834292; doi:10.1371/journal.pcbi.1007357)

Maximum likelihood estimate of the covariance matrix

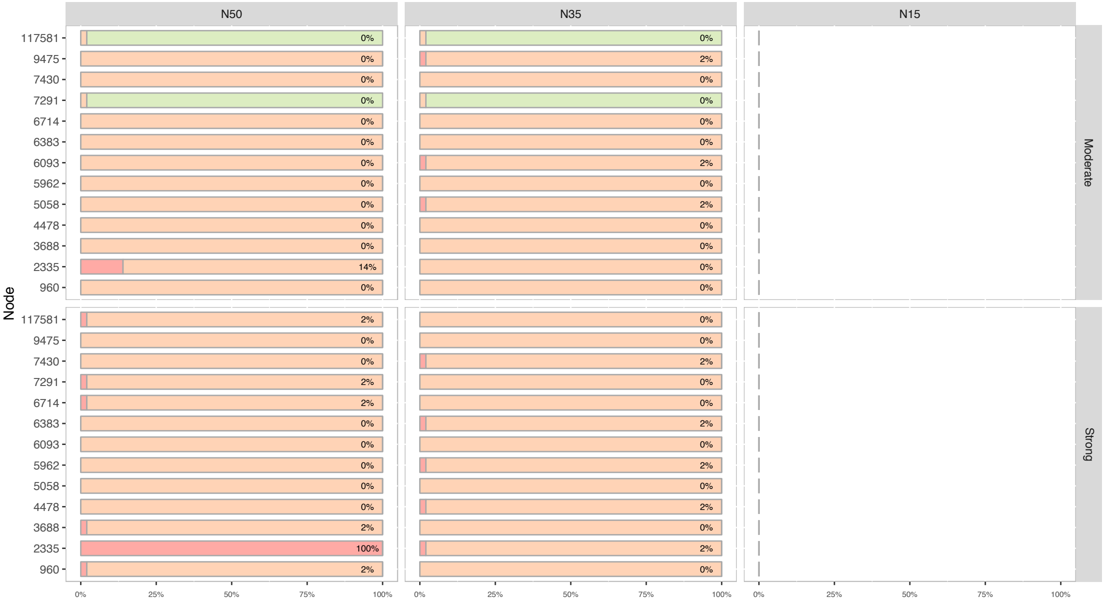

Regularized estimate of the covariance matrix

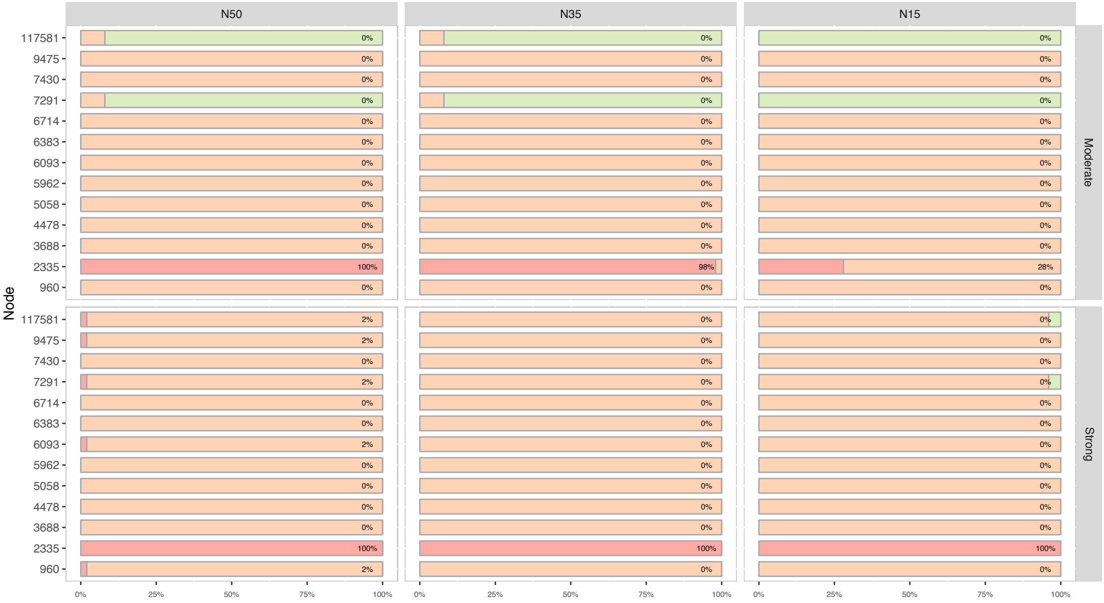

Supplement: S2 Fig — On the top panel, results based on the maximum likelihood estimate of the covariance matrix; on the bottom panel results based on the regularized estimate. Each subpanel corresponds to a different combination of the sample size (columns) and the intensity of dysregulation (rows). Inside subpanels, for each variable Xv, v ∈ V a stacked bar chart shows the percentages of times that v∈D^G (red, primary set), v∈D^G\D^G (orange, secondary set) and v∈V\D^G (green). Only genes that appear at least one time in the source set are shown (13 out of 202). Two subpanels are missing because of the maximum likelihood estimate does not exit (i.e., n ≤ p*). (PDF) [file pcbi.1007357.s009.pdf]
