# Supplementary figures and images for: SourceSet: A graphical model approach to identify primary genes in perturbed biological pathways
Source: PLoS Comput Biol. 2019 Oct 25;15(10):e1007357. doi: 10.1371/journal.pcbi.1007357 (PMC6834292; doi:10.1371/journal.pcbi.1007357)

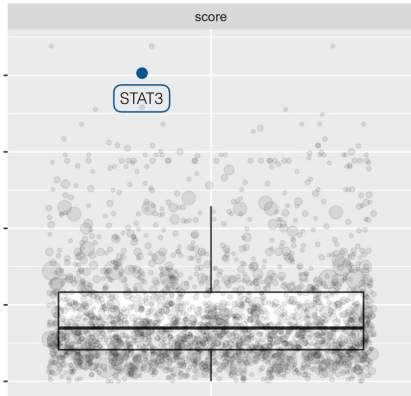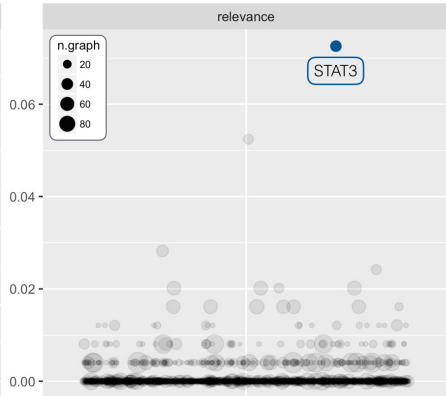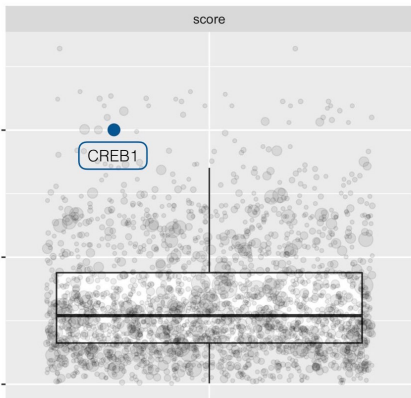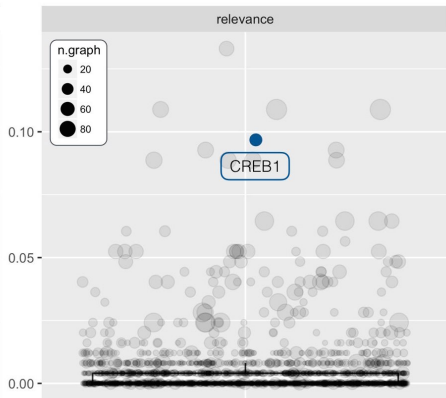

Supplement: S3 Fig — Boxplots of score (left panel) and relevance (right panel) indices for genes annotated in at least two pathways of the whole KEGG collection (N = 248). The size of each point is proportional to the number of pathways in which the associated gene is annotated. Silenced or knock-down genes are highlighted with blue dots. For more details about the interpretation of each index, see S7 Text. (PDF) [file pcbi.1007357.s010.pdf]

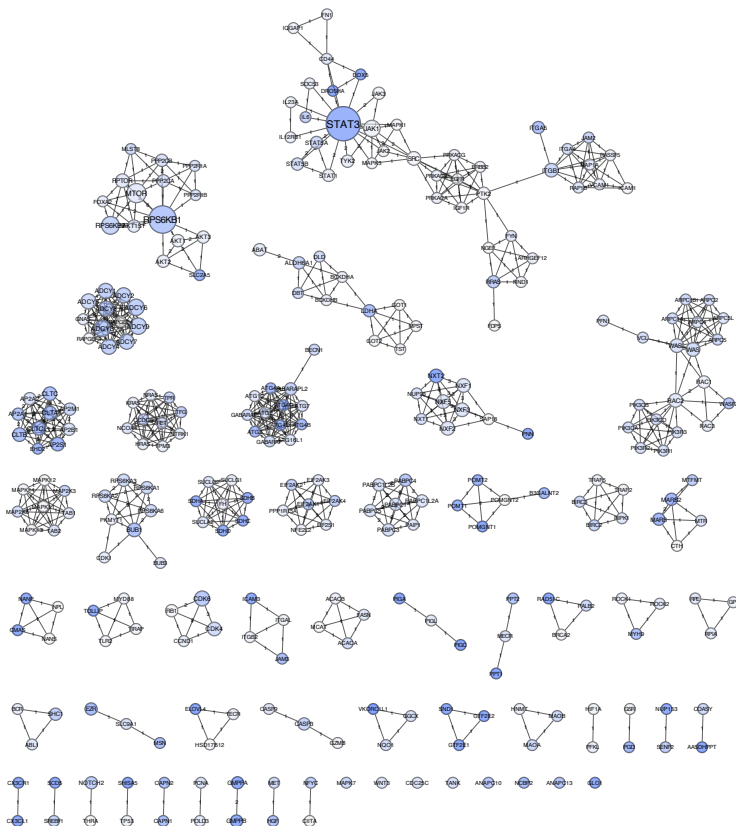

Supplement: S4 Fig — The graphical union of all subgraphs induced by source set elements of each analyzed pathway (N = 248) is represented. The size of each node is proportional to the number of times the gene appears in a source set. The color is associated with the score index: higher values are highlighted with darker blue color. The number depicted on each edge represents the number of pathways in which the two genes are connected. For more details about the interpretation of each index, see S7 Text. (PDF) [file pcbi.1007357.s011.pdf]

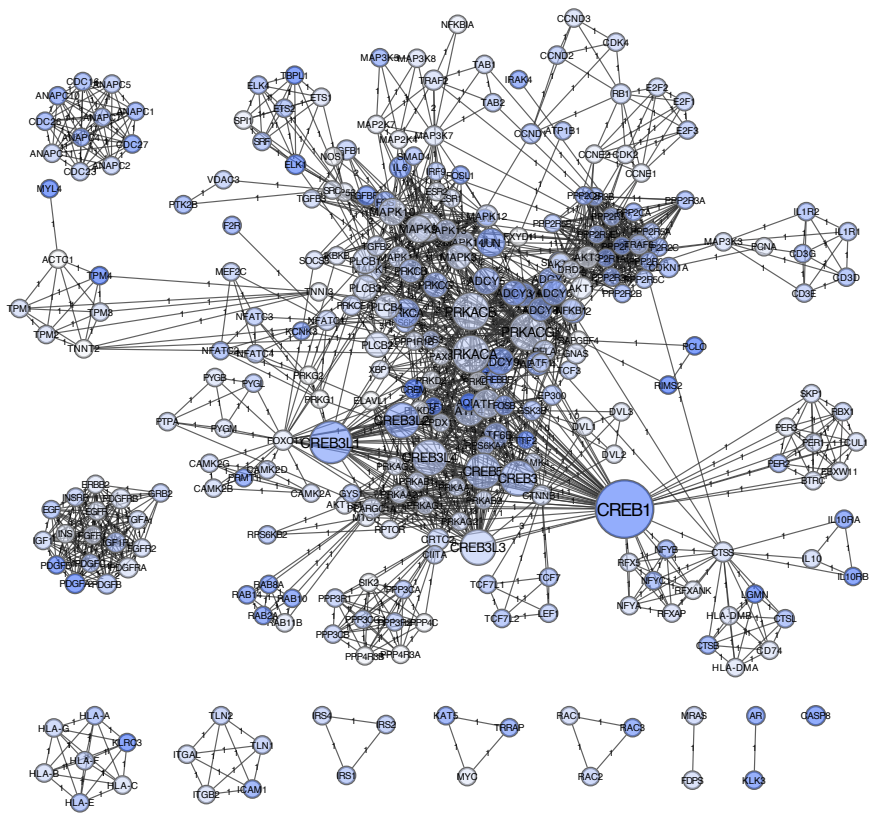

Supplement: S5 Fig — The graphical union of all subgraphs induced by source set elements of each analyzed pathway (N = 248) is represented. The size of each node is proportional to the number of times the gene appears in a source set. The color is associated with the score index: higher values are highlighted with darker blue color. The number depicted on each edge represents the number of pathways in which the two genes are connected. For more details about the interpretation of each index, see S7 Text. (PDF) [file pcbi.1007357.s012.pdf]

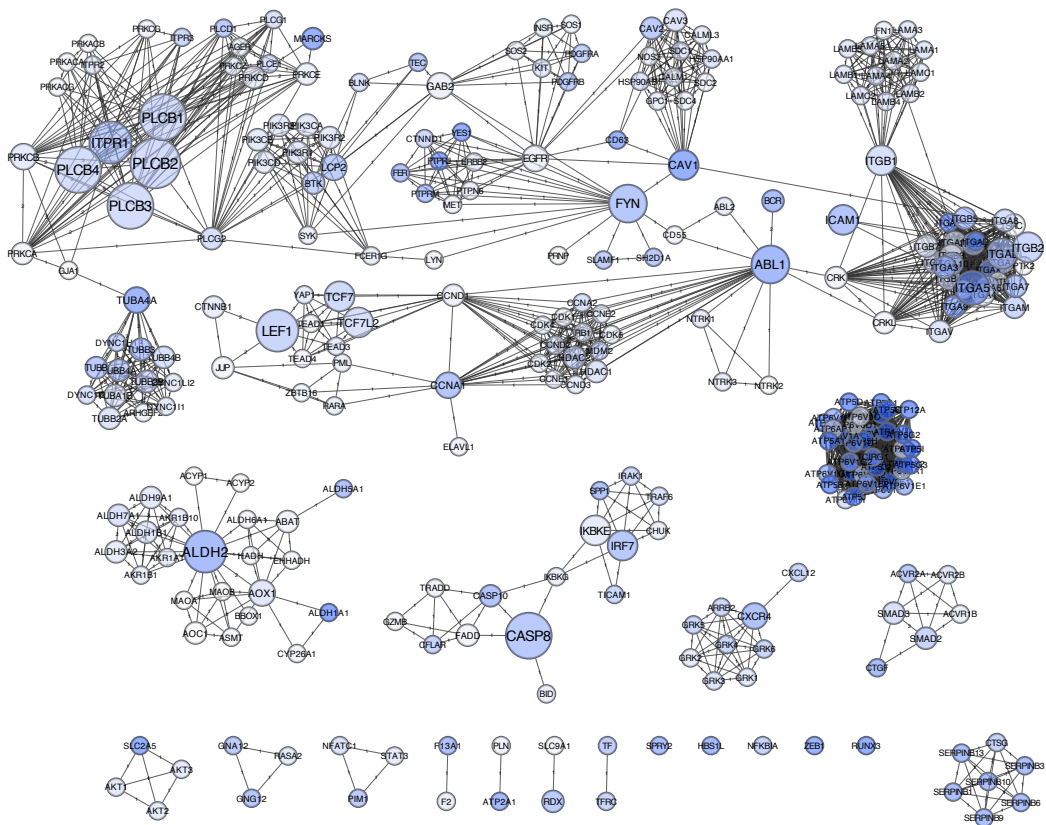

Supplement: S6 Fig — The graphical union of all subgraphs induced by source set elements of each analyzed pathway (N = 248) is represented. The size of each node is proportional to the number of times the gene appears in a source set. The color is associated with the score index: higher values are highlighted with darker blue color. The number depicted on each edge represents the number of pathways in which the two genes are connected. For more details about the interpretation of each index, see S7 Text. (PDF) [file pcbi.1007357.s013.pdf]
