# Supplementary material for: SourceSet: A graphical model approach to identify primary genes in perturbed biological pathways
Source: PLoS Comput Biol. 2019 Oct 25;15(10):e1007357. doi: 10.1371/journal.pcbi.1007357 (PMC6834292; doi:10.1371/journal.pcbi.1007357)
Supplement: S1 Table — Pathways in which the silenced gene appears in the source set are marked with a star. In particular those in which the silenced gene is the only gene of the source set are highlighted in gray. For more details about the interpretation of each index, see S7 Text. (PDF) [file pcbi.1007357.s014.pdf]

		$\lvert\hat{D}_G\rvert$	$\lvert\hat{\mathbb{D}}_{\mathbf{G}} \setminus \hat{D}_G\rvert$	$\lvert V\rvert$	n.cluster	primary impact	total impact	pvalue
$\star$	Acute myeloid leukemia	5	9	55	1	0.091	0.255	<0.001
$\star$	Adipocytokine signaling pathway	1	27	62	1	0.016	0.452	<0.001
$\star$	AGE-RAGE signaling pathway ...	1	14	87	1	0.011	0.172	<0.001
$\star$	EGFR tyrosine kinase inhibitor ...	6	20	79	1	0.076	0.329	<0.001
$\star$	Epstein-Barr virus infection	14	12	81	7	0.173	0.321	<0.001
	FoxO signaling pathway	0	37	120	2	0.000	0.308	<0.001
$\star$	Hepatitis B	8	4	129	4	0.062	0.093	<0.001
$\star$	HIF-1 signaling pathway	3	12	97	1	0.031	0.155	<0.001
$\star$	Inflammatory bowel disease (IBD)	3	9	47	1	0.064	0.255	<0.001
$\star$	Insulin resistance	3	18	91	1	0.033	0.231	<0.001
$\star$	Measles	3	0	99	9	0.030	0.030	<0.001
$\star$	MicroRNAs in cancer	9	0	136	136	0.066	0.066	<0.001
$\star$	Non-small cell lung cancer	2	0	56	2	0.036	0.036	<0.001
$\star$	Pancreatic cancer	4	15	62	4	0.065	0.306	<0.001
	Pathways in cancer	0	9	304	3	0.000	0.030	<0.001
$\star$	Prolactin signaling pathway	4	35	70	1	0.057	0.557	0.001
$\star$	Proteoglycans in cancer	8	44	195	3	0.041	0.267	<0.001
$\star$	Signaling pathways regulating ...	1	14	107	1	0.009	0.140	<0.001
$\star$	Th17 cell differentiation	1	38	90	2	0.011	0.433	<0.001
$\star$	Toxoplasmosis	3	6	87	6	0.034	0.103	<0.001
