# Supplementary material for: SourceSet: A graphical model approach to identify primary genes in perturbed biological pathways
Source: PLoS Comput Biol. 2019 Oct 25;15(10):e1007357. doi: 10.1371/journal.pcbi.1007357 (PMC6834292; doi:10.1371/journal.pcbi.1007357)
Supplement: S2 Table — Pathways in which the knock-down gene appears in the source set are marked with a star. In particular, those in which CREB is the only source set element are highlighted in gray. For more details about the interpretation of each index, see S7 Text. (PDF) [file pcbi.1007357.s015.pdf]

		$\lvert\hat{D}_G\rvert$	$\lvert\hat{\mathbb{D}}_{\mathbf{G}} \setminus \hat{D}_G\rvert$	$\lvert V\rvert$	n.cluster	primary impact	total impact	pvalue
⋆	Adrenergic signaling in cardio...	16	68	144	1	0.111	0.583	<0.001
⋆	Aldosterone synthesis and secretion	34	17	65	2	0.523	0.785	<0.001
⋆	Amphetamine addiction	13	21	62	2	0.210	0.548	<0.001
⋆	AMPK signaling pathway	29	15	94	3	0.309	0.468	<0.001
⋆	Antigen processing and presentation	16	0	62	4	0.258	0.258	<0.001
⋆	cAMP signaling pathway	18	60	193	1	0.093	0.404	<0.001
⋆	cGMP-PKG signaling pathway	13	38	153	1	0.085	0.333	<0.001
⋆	Cholinergic synapse	12	22	95	1	0.126	0.358	<0.001
⋆	Circadian entrainment	8	48	96	1	0.083	0.583	<0.001
⋆	Circadian rhythm	9	0	31	1	0.290	0.290	0.001
⋆	Cocaine addiction	17	7	42	1	0.405	0.571	<0.001
⋆	Dopaminergic synapse	36	23	124	1	0.290	0.476	<0.001
⋆	Estrogen signaling pathway	11	20	87	1	0.126	0.356	<0.001
⋆	Glucagon signaling pathway	25	21	85	1	0.294	0.541	<0.001
	Hepatitis B	21	59	132	3	0.159	0.606	<0.001
⋆	HTLV-I infection	73	35	190	10	0.384	0.568	<0.001
⋆	Insulin resistance	22	50	92	1	0.239	0.783	<0.001
⋆	Insulin secretion	32	4	53	2	0.604	0.679	<0.001
	Melanogenesis	17	22	100	2	0.170	0.390	<0.001
⋆	Osteoclast differentiation	22	27	118	2	0.186	0.415	<0.001
⋆	Prostate cancer	46	16	85	1	0.541	0.729	<0.001
⋆	Renin secretion	1	6	50	4	0.020	0.140	0.001
⋆	Thyroid hormone synthesis	26	11	46	2	0.565	0.804	<0.001
⋆	TNF signaling pathway	16	17	70	1	0.229	0.471	<0.001
⋆	Tuberculosis	26	50	173	4	0.150	0.439	<0.001
⋆	Vasopressin-regulated water ...	14	1	22	2	0.636	0.682	<0.001
