# Supplementary material for: SourceSet: A graphical model approach to identify primary genes in perturbed biological pathways
Source: PLoS Comput Biol. 2019 Oct 25;15(10):e1007357. doi: 10.1371/journal.pcbi.1007357 (PMC6834292; doi:10.1371/journal.pcbi.1007357)
Supplement: S3 Table — Pathways in which ABL1 or both chimera genes appear in the source set are marked with one or two stars, respectively. In particular, those in which ABL1 and BCR are the only elements of the source set are highlighted in gray. For more details about the interpretation of each index, see S7 Text. (PDF) [file pcbi.1007357.s016.pdf]

		$\lvert\hat{D}_G\rvert$	$\lvert\hat{\mathbb{D}}_{\mathbf{G}} \setminus \hat{D}_G\rvert$	$\lvert V\rvert$	n.cluster	primary impact	total impact	pvalue
	Axon guidance	0	44	126	2	0.000	0.349	0.016
⋆	Cell cycle	16	10	111	1	0.144	0.234	0.011
⋆⋆	Chronic myeloid leukemia	2	30	67	3	0.030	0.478	0.016
⋆	ErbB signaling pathway	3	9	78	1	0.038	0.154	0.067
⋆	Neurotrophin signaling pathway	4	8	98	2	0.041	0.122	0.014
⋆⋆	Pathways in cancer	3	50	263	4	0.011	0.202	0.003
⋆	Viral myocarditis	8	5	25	6	0.320	0.520	$<$0.001
