# Supplementary material for: SourceSet: A graphical model approach to identify primary genes in perturbed biological pathways
Source: PLoS Comput Biol. 2019 Oct 25;15(10):e1007357. doi: 10.1371/journal.pcbi.1007357 (PMC6834292; doi:10.1371/journal.pcbi.1007357)
Supplement: S4 Table — infoSource summary for the top five genes ordered by relevance index. Number of analyzed pathways in which the gene belongs to the primary dysregulation (n.primary) or the secondary dysregulation (n.secondary); number of analyzed pathways in which it is annotated (n.graph), and its score and relevance indices. For more details about the interpretation of each index, see S7 Text. (PDF) [file pcbi.1007357.s017.pdf]

Symbol	n.primary	n.secondary	n.graph	score	relevance
RAF1	17	42	57	1.602	0.069
ARAF	17	26	27	2.564	0.069
STAT3	12	20	20	2.174	0.048
TP53	9	24	28	1.833	0.036
MAP2K1	7	38	63	1.588	0.028
