# Supplementary material for: SourceSet: A graphical model approach to identify primary genes in perturbed biological pathways
Source: PLoS Comput Biol. 2019 Oct 25;15(10):e1007357. doi: 10.1371/journal.pcbi.1007357 (PMC6834292; doi:10.1371/journal.pcbi.1007357)
Supplement: S5 Table — infoSource summary for the top 10 pathways, ordered by decreasing primary.impact. For more details about the interpretation of each index, see S7 Text. (PDF) [file pcbi.1007357.s018.pdf]

	$\lvert\hat{D}_G\rvert$	$\lvert\hat{\mathbb{D}}_{\mathbf{G}} \setminus \hat{D}_G\rvert$	$\lvert V\rvert$	n.cluster	primary impact	total impact	pvalue
Mucin type O-glycan biosynthesis	15	1	16	1	0.938	1.000	<0.001
Bile secretion	8	0	11	2	0.727	0.727	0.015
Ether lipid metabolism	14	6	21	1	0.667	0.952	0.013
Steroid hormone biosynthesis	11	3	17	2	0.647	0.824	<0.001
Tyrosine metabolism	8	0	14	2	0.571	0.571	<0.001
Sphingolipid metabolism	20	0	36	1	0.556	0.556	0.010
beta-Alanine metabolism	12	2	22	2	0.545	0.636	<0.001
Retinol metabolism	8	6	16	1	0.500	0.875	<0.001
Primary bile acid biosynthesis	3	0	7	2	0.429	0.429	0.029
Vibrio cholerae infection	5	2	12	3	0.417	0.583	<0.001
