# Supplementary material for: SourceSet: A graphical model approach to identify primary genes in perturbed biological pathways
Source: PLoS Comput Biol. 2019 Oct 25;15(10):e1007357. doi: 10.1371/journal.pcbi.1007357 (PMC6834292; doi:10.1371/journal.pcbi.1007357)
Supplement: S6 Table — infoSource summary for the top five genes—annotated in at least 2 pathways—ordered by score index. Number of analyzed pathways in which the gene belongs to the primary dysregulation (n.primary) or the secondary dysregulation (n.secondary); number of analyzed pathways in which it is annotated (n.graph), and its score and relevance indices. For more details about the interpretation of each index, see S7 Text. (PDF) [file pcbi.1007357.s019.pdf]

Symbol	n.primary	n.secondary	n.graph	score	relevance
HSD17B6	2	0	2	5.059	0.0081
GHR	2	0	2	4.025	0.0081
GRIA2	6	1	7	3.734	0.0242
HLA-DMB	4	0	5	3.562	0.0161
COMT	2	0	2	3.506	0.0081
