# Supplementary material for: SourceSet: A graphical model approach to identify primary genes in perturbed biological pathways
Source: PLoS Comput Biol. 2019 Oct 25;15(10):e1007357. doi: 10.1371/journal.pcbi.1007357 (PMC6834292; doi:10.1371/journal.pcbi.1007357)
Supplement: S7 Table — Precision and type I error (in parentheses) in different simulation settings. For non-sequential mapggm procedure the precision is computed as the probability that node 5 is first-ranked and significant; type I error is calculated as the probability that another node—other than node 5—is first-ranked with a significant p-value. (PDF) [file pcbi.1007357.s020.pdf]

	Mapggm		
	$n = 5$	$n = 10$	$n = 25$
Weak	0.07 (0.37)	0.10 (0.22)	0.22 (0.09)
Mild	0.76 (0.20)	0.98 (0.02)	1.00 (< 0.01)
Moderate	0.93 (0.07)	1.00 (< 0.01)	1.00 (< 0.01)
Strong	0.97 (0.03)	1.00 (< 0.01)	1.00 (< 0.01)
